# Divergence With Gene Flow and Contrasting Population Size Blur the Species Boundary in *Cycas* Sect. *Asiorientales*, as Inferred From Morphology and RAD-Seq Data

**DOI:** 10.3389/fpls.2022.824158

**Published:** 2022-05-09

**Authors:** Jui-Tse Chang, Chien-Ti Chao, Koh Nakamura, Hsiao-Lei Liu, Min-Xin Luo, Pei-Chun Liao

**Affiliations:** ^1^School of Life Science, National Taiwan Normal University, Taipei, Taiwan; ^2^Botanic Garden, Field Science Center for Northern Biosphere, Hokkaido University, Sapporo, Japan; ^3^Department of Anthropology, Smithsonian Institution, National Museum of Natural History, Washington, DC, United States

**Keywords:** continental island, *Cycas*, Kuroshio, long distance dispersal, speciation, species concept

## Abstract

The divergence process of incipient species is fascinating but elusive by incomplete lineage sorting or gene flow. Species delimitation is also challenging among those morphologically similar allopatric species, especially when lacking comprehensive data. *Cycas* sect. *Asiorientales*, comprised of *C. taitungensis* and *C. revoluta* in the Ryukyu Archipelago and Taiwan, diverged recently with continuous gene flow, resulting in a reciprocal paraphyletic relationship. Their previous evolutionary inferences are questioned from few genetic markers, incomplete sampling, and incomprehensive morphological comparison by a long-term taxonomic misconception. By whole range sampling, this study tests the geographic mode of speciation in the two species of *Asiorientales* by approximate Bayesian computation (ABC) using genome-wide single nucleotide polymorphisms (SNPs). The individual tree was reconstructed to delimit the species and track the gene-flow trajectory. With the comparison of diagnostic morphological traits and genetic data, the allopatric speciation was rejected. Alternatively, continuous but spatially heterogeneous gene flow driven by transoceanic vegetative dispersal and pollen flow with contrasting population sizes blurred their species boundary. On the basis of morphological, genetic, and evolutionary evidence, we synonymized these two *Cycas* species. This study highlights not only the importance of the Kuroshio Current to species evolution but also the disadvantage of using species with geographically structured genealogies as conservation units.

## Introduction

Incipient species are critical for evolutionary biologists to study speciation, but they also challenge taxonomy due to gene flow or ancestral polymorphism. The former and contrasting population size lead to larger intraspecific than interspecific variations, a phenomenon called the species-definition anomaly zone (Jiao and Yang, 2021). The latter results in inconsistent species delimitation and gene tree, called the species-tree anomaly zone (Degnan and Rosenberg, 2006). Because speciation is a continuous process, various species concepts that impose discrete (sub)species status also confuses taxonomic status. Currently, the unified species concept (USC), one of 34 species concepts (Zachos, 2018), integrates species as separately evolving metapopulations and considers other species concepts as operational criteria to mitigate the problem (but also see Freudenstein et al. (2017), for the “phenophyletic view” of the species concept).

*Cycas* L. is a taxonomically complex group because of the recent divergence of extant taxa in the Paleocene, morpho-ecological conservatism, and the fragmented geographic distribution (Norstog and Nicholls, 1997; Nagalingum et al., 2011; Xiao and Moller, 2015; Zheng et al., 2017; Habib et al., 2021; Liu et al., 2021b). These factors make *Cycas* a sound study system for the geographic mode of speciation. The mainstream geography-of-speciation research has focused on gene flow patterns during speciation. However, this approach is inadequate without considering the distribution and range size to distinguish allopatric from non-allopatric speciation (Hernandez-Hernandez et al., 2021). Further, next-generation sequencing (NGS) techniques such as restriction site-associated DNA (RAD) improve the inferences of speciation processes (Maguilla et al., 2017; Nikolic et al., 2020; Goodall et al., 2021). Estimating allopatric-to-non-allopatric speciation frequencies is important to learn the community assembly process and evolutionary and ecological mechanisms of biodiversity (Skeels and Cardillo, 2019; García-Navas et al., 2020). However, considerations on the geographic mode of speciation are scarce in recent *Cycas* studies (Liu et al., 2015; Feng et al., 2016, 2021; Wang et al., 2019). Although most systematic studies conducted integrative taxonomy following the USC, few genetic markers were used, making them questionable given the porous and semipermeable features of the genome (Wu, 2001; Harrison, 2012; Wolf and Ellegren, 2017).

**FIGURE 1:**
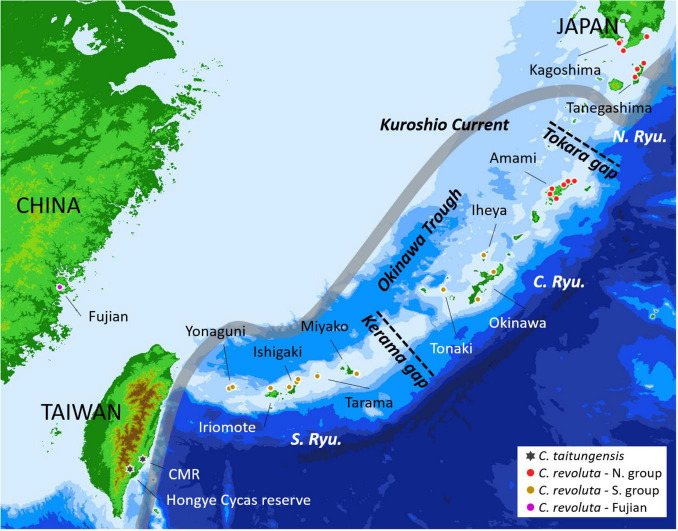
Sampling localities of *Cycas revoluta and C. taitungensis* for double-digest restriction site-associated DNA sequencing. The gray route is the course of the Kuroshio Current. The red and brown colors denote northern and southern groups according to the morphogenetic structure. CMR: Coastal Mountain Range; N. Ryu, C. Ryu, and S. Ryu refer to the northern, central, and southern Ryukyus, respectively.

*Cycas* sect. *Asiorientales* comprises *C. taitungensis* C. F. Shen et al. (1994) and *C. revoluta* Thunb. The former is endemic to Taiwan with two small populations; the latter is broadly distributed in southern Kyushu, and the Ryukyu Archipelago, Japan, and a small population in Fujian, China, with large but fragmented populations (Liu et al., 2018; Figure 1). The Ryukyu Archipelago and Taiwan are at the continental margin, and the land configuration has changed drastically to become fragmented. They have been reconnected over time through crustal movements and paleoclimatic sea-level changes (Wang et al., 2014). The Kerama and Tokara Gaps are two major geological gaps dividing the Ryukyu Archipelago into the northern, central, and southern groups (Figure 1). For species with limited dispersibility like *C. revoluta* and *C. taitungensis* (Dehgan and Yuen, 1983; Kono and Tobe, 2007), these landscape dynamics may influence their population genetic structure and evolutionary trajectories. Previous studies of *C. revoluta* have shown that morphogenetic variations correspond to geography (Setoguchi et al., 2009; Kyoda and Setoguchi, 2010). Compared with *C. revoluta*, *C. taitungensis* exhibits high genetic diversity despite the small census population size (Chiang et al., 2009; Zheng et al., 2017; Chang et al., 2019). These two sister species are in the stage of incipient speciation, showing a species tree anomaly zone based on different markers (Chiang et al., 2009, 2018; Chang et al., 2019). The high ancestral polymorphism and historical gene flow further complicate the study of their speciation (Chiang et al., 2009, 2018; Smith et al., 2018; Chang et al., 2019). Although the southward founder speciation of *C. taitungensis* has been inferred (Chang et al., 2019), the few dominant markers, incomplete sampling, and ignorance of different gene flow possibilities would mask or even bias the inference, for example, miscalculating the heterozygotes or mistaking the intraspecific and interspecific variations, especially for those with large genomes (mean size = 26.2 pg/2C in *Cycas*) (Funk and Omland, 2003; Zonneveld, 2011).

Taxonomically, *C. taitungensis* had long been mistaken for *C. taiwaniana* Carruth. in section *Stangerioides* (Shen et al., 1994). Due to the taxonomic misconception, the morphological comparison between *C. taitungensis* and *C. revoluta* is lacking or erroneous, even in the protolog of *C. taitungensis* (Shen et al., 1994). Shen et al. (1994) did not compare characters between the two species but just adopted the problematic comparison of Yamamoto (1928) (see taxonomic status below). Other references have distinguished *C. revoluta* from *C. taitungensis* by the following characters in the latter species: (1) shorter, narrower, and keeled leaves, and leaflets; (2) strongly recurved or revolute leaflet margins; (3) more tightly imbricate female cones; (4) smaller and ovate (vs. elliptical) seeds with darker sarcotesta and non-grooved sclerotesta; and (5) ovate to lanceolate megasporophyll terminal lamella (Wang, 1996; De Laubenfels and Adema, 1998; Chen and Stevenson, 1999; Whitelock, 2002; Hill, 2008). However, the compared specimens were mostly from restricted areas and did not take into account comprehensive intraspecific variation.

To understand the speciation process and clarify comprehensive genetic variations, we applied genome-wide double-digest RAD sequencing (ddRAD-seq) alongside broad-range sampling. Morphometrics with a focus on diagnostic traits was also evaluated. We aimed to (1) clarify the population genetic structure across the whole distribution range of sect. *Asiorientales*, (2) infer the geographic mode of speciation in *C. revoluta* and *C. taitungensis*, and (3) reappraise the species status in sect. *Asiorientales*. For the geographic mode of speciation, the allopatric and non-allopatric speciation hypotheses were tested. The former expected no gene flow during speciation, and the divergence time may correspond to the submergence of land bridges in the Ryukyu Archipelago-Taiwan. In contrast, gene flow can still be detected during species divergence if speciation was not driven by geographic isolation, and the divergence time may be dated in prevalent land bridges in the Ryukyu Archipelago-Taiwan. The gene flow and their divergence time will be estimated using the coalescence-based method, and the hypotheses will be tested *via* model selection under the Bayesian method.

## Materials and Methods

The packages for analyses were all conducted in R version 3.6.1 (R Core Team, 2015).

### Population Sampling for ddRAD-Seq

We sampled 26 populations of *C. revoluta* (*n* = 209) in the Ryukyu Archipelago and southern Kyushu in Japan, the Fujian Province in China, and two populations of *C. taitungensis* (*n* = 30) in Taiwan (Figure 1 and Supplementary Table 1). The numbers of sampling populations and individuals were different between the two species but inevitably biased due to the small census population size and merely two populations of *C. taitungensis*. Although the removal of siblings would inflate the genetic diversity and effective population size (Hendricks et al., 2018), the frequent vegetative bulbils fallen from *C. revoluta* may inversely underestimate these indices. Therefore, the individuals separated by at least 5 m were sampled to prevent the replication from asexual individuals. Also, we only selected the populations growing in clusters, and hence, the information from nearby siblings would be retained. Fresh leaflets were dried in silica gel and preserved at 4°C for DNA extraction.

### Bioinformatics of Single Nucleotide Polymorphism Calling

Genomic DNA was extracted following a modified protocol from Doyle (1991). Genomic DNA quality and quantity were checked using a Qubit 2.0 fluorometer (Life Technologies, Carlsbad, CA, United States). The reduced-representation ddRAD approach was used to acquire reliable and high-coverage biallelic single nucleotide polymorphism (SNPs). The qualified DNA was double digested by *Sbf*1 (recognition site: 5′-CCTGCAGG-3′) and *Msp*1 (5′-GGCC-3′) and ligated to Illumina adapters with individual barcodes and library indices. Following the library construction protocol of Peterson et al. (2012) with size selection targeting 250–500 bp, 150-bp paired-end reads were sequenced in 12 libraries using the Illumina HiSeq X Ten (Illumina, San Diego, CA, United States).

The raw reads were checked by multiQC with a Phred score > 30 before *de novo* mapping. Stacks 2.2 (Catchen et al., 2013) was used to optimize the parameters in *ustacks* (*M* and *n* from 1 to 6) and *cstacks* (*m* from 3 to 6) considering the error rate, locus number, and coverage optimization following the methods of Mastretta-Yanes et al. (2015). The dataset was processed with a *de novo* pipeline with *M* = *n* = 6 and *m* = 3 without predefined groups. A total of 57 samples (*N* = 45 and 12 for *C. revoluta* and *C. taitungensis*, respectively) were used to construct catalogs in *gstacks*. Of the resulting SNPs, only one was retained in each RAD locus to decrease the linkage effect, and sites with a missing rate > 0.4 (*p* = 1, *r* = 0.4), a minor allele frequency (MAF) < 0.01 (–min–maf = 0.01), and maximum heterozygosity > 0.8 (–max–obs = 0.8) were excluded in the populations program. The PCR duplicates had little effect on genotype calling and heterozygote in high-quality DNA and sequencing data (Supplementary Table 2) and were therefore not removed (Euclide et al., 2020). Unlinked SNPs with a mean allele depth < 5, missing rate > 0.55, and individual missing rate > 0.5 were removed by Vcftools (Danecek et al., 2011).

### Population Structure

To understand the population genetic structure of the whole distribution range, the model-free principal coordinate analysis (PCoA) and model-based ancestry coefficient estimations were performed. Nei’s distance matrix among sampling populations was transformed by nei.dist function in the poppr package (Kamvar et al., 2014) and input for PCoA in the cmdscale function from the stats package.

**FIGURE 2:**
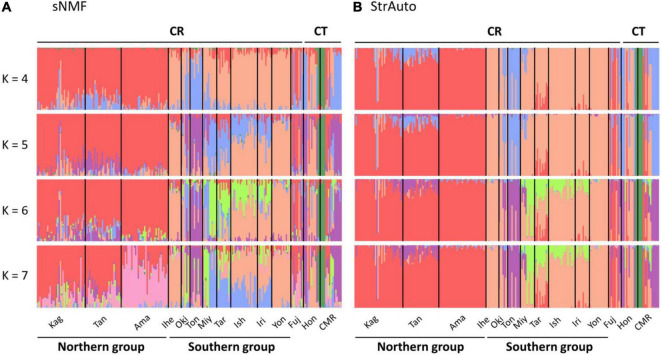
Island population genetic structure inferred by **(A)** the sparse nonnegative matrix factorization (sNMF) algorithm and **(B)** the StrAuto software. CR and CT denote *Cycas revoluta* and *Cycas taitungensis*, respectively. The island names are abbreviated as the first three characters of the full names.

The ancestry coefficient was estimated without a predefined group to clarify the intra-section structure. There was a total of 14 populations in our study (Figure 2), and we assumed at most one population contained its specific structure. Hence, *K* was set from 1 to 15 in StrAuto 0.3.1 (Chhatre, 2012) and the sparse nonnegative matrix factorization (sNMF) algorithm in the snmf function of the LEA package (Frichot et al., 2015). The former analysis was optimized by 100,000 iterations with 10,000 burn-in, and the consensus was reached with 10 independent runs; the latter was optimized by 1,000 iterations, and the consensus was also reached with 10 independent runs. Evanno’s method in CLUMPAK (Kopelman et al., 2015) and cross-entropy were used to validate the best ancestral proportions (i.e., *K*) in StrAuto and sNMF, respectively.

Population clustering exhibited larger intraspecific than interspecific variations, corresponding to the geographic distribution (see Results). Consequently, the boundaries and strengths of genetic barriers were estimated considering the sampling locality connections and Nei’s genetic distances in the adegenet 1.3–1 package (Jombart and Ahmed, 2011). Three connection networks were drawn by chooseCN function to represent the intensive [Delaunay triangulation (DT)], moderate [nearest neighbor (NN)], and low [minimum spanning tree (MP)] connection strength. Particularly for NN, due to the lack of dispersibility for sect. *Asiorientales*, *K* was set to 7 to consider the connections of a quarter of the sampling (i.e., *N*/4 = 28/4). The barriers were searched by 20 optimizations with the optimize.monmonier function and the local differences were set to zero to search possible boundaries. The island-level pairwise *F*st values were also computed to quantify the divergence patterns using arlsumstat in the ABCtoolbox (Wegmann et al., 2010).

### Speciation Modeling of *Cycas revoluta* and *Cycas taitungensis*

**FIGURE 3:**
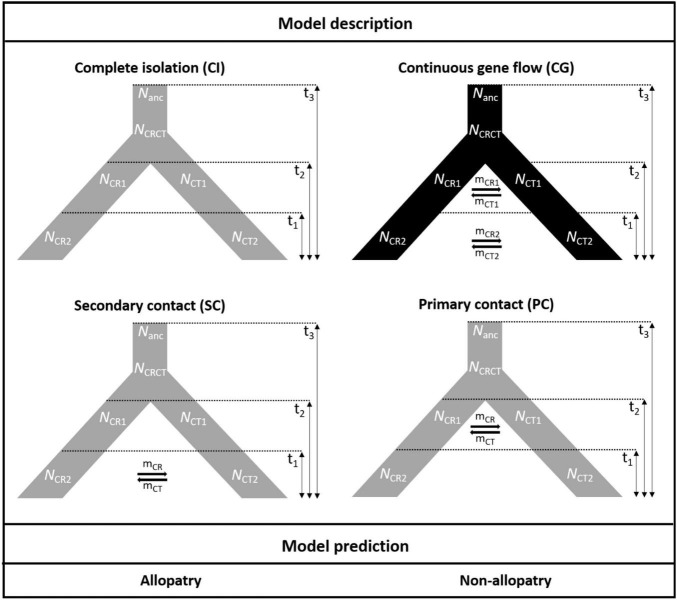
The four speciation models testing the demography and gene flow patterns in the approximate Bayesian computation models. The parameters are defined in Table 1.

To test the hypotheses of the geographic mode of speciation, the approximate Bayesian computation (ABC) was conducted. Because of the different range sizes between the two cycads, four speciation models were proposed to consider gene flow and demography. Scenarios combining gene flow and demographic changes were set to fit the poor transoceanic dispersibility of these two species (Dehgan and Yuen, 1983; Kono and Tobe, 2007). Based on Coyne and Orr (2004), allopatric speciation is defined as complete geographic isolation without gene flow. Due to the lack of fossil records to track back the paleodistribution of the onset of speciation, allopatric, and non-allopatric speciation was determined by the absence and presence of gene flow in the incipient speciation stages, respectively (also see Dong et al., 2020). We also accounted for the post-divergence gene flow by secondary contact. Overall, the non-allopatric speciation models were (1) speciation with continuous gene flow (CG) and (2) primary contact (PC); the allopatric speciation models were (3) speciation with complete isolation (CI) and (4) secondary contact (SC). In parameter settings, the divergence of the two species was set < 9 mya based on Nagalingum et al. (2011) and transferred to 3 × 10^5^ generations considering 30 years per generation. There are several records for the *Cycas* generation time, from more than 10 to 40 years in the International Union for Conservation of Nature (IUCN; Mankga and Yessoufou, 2017). Because the IUCN generation time records are rough and lack detailed justifications (Zheng et al., 2017), and *C. taitungensis* has been recorded to grow fast—5 cm annually (Huang et al., 2004)—we have conservatively taken 30 years for the *Cycas* generation time (Stewart and Globig, 2011; Table 1 and Figure 3; see Supplementary Table 3 and Supplementary Method 1 for prior settings and detailed model description).

**TABLE 1:** Description of the model parameters in the four speciation models.

Parameter	Description
N_CR2_	Extant effective population size in *Cycas revoluta*
N_CT2_	Extant effective population size in *Cycas taitungensis*
N_anc_REL_	Relative effective population size of ancestral sect. *Asiorientales*
N_CR_REL_	Relative effective population size of *C. revoluta* before t1 (i.e., N_CR1_)
N_CT_REL_	Relative effective population size of *C. taitungensis* before t1 (i.e., N_CT1_)
t1	Time of migration and demography change between *C. revoluta* and *C. taitungensis*
t2	Divergence time of *C. revoluta* and *C. taitungensis*
t3	Divergence time of sect. *Asiorientales* and its sister
m_CT1_	Migration rate of *C. taitungensis* to *C. revoluta* per generation before t1
m_CR1_	Migration rate of *C. revoluta* to *C. taitungensis* per generation before t1
m_CT2_	Migration rate of *C. taitungensis* to *C. revoluta* per generation after t1
m_CR2_	Migration rate of *C. revoluta* to *C. taitungensis* per generation after t1
μ	Mutation rate in sect. *Asiorientales*

One million simulations for four divergence models were implemented in fastsimcoal 2 (Excoffier et al., 2013), and 20 summary statistics (i.e., species-specific segregating sites; number of pairwise differences; *He* and its standard deviation; global mean and standard deviation of segregating sites, number of pairwise differences, *F*_st_, and *F*_*it*_; interspecific *F*_st_) were computed using arlsumstat in ABCtoolbox (Wegmann et al., 2010). Posterior probability was performed using the best 1,000 simulations after removing highly correlated summary statistics for the model selection by the Bayes factor (Jeffreys, 1998) in the ABCtoolbox (see Supplementary Method 1 for detailed quality control and validations before model selection). The precision of the best model on the observed data was quantified by the goodness-of-fit test. Parameters of the best model were log-transformed and estimated by retained summary statistics by 15 hidden layers and 50 feed-forward neural networks in non-linear regression using the abc function in abc package (Csilléry et al., 2012).

### Population and Individual Phylogeny Reconstructions

Since frequent gene flow was detected between these two cycads (see “Results”), we re-delineated the species boundary using the phylogenetic analysis. The transformed Nei’s distance was used to reconstruct the neighbor-joining tree with 1,000 bootstraps by the aboot function in poppr package (Kamvar et al., 2014). Because of apparent genetic admixture, the individual tree was further reconstructed to detail the intraspecific relationship for species delimitation.

To reduce the intensive computation, we selected 110 individuals (6–9 individuals per island) with low individual missing rate (range from 16 to 50%) according to their genetic components. For *C. taitungensis*, the cryptic lineage (i.e., the green genetic component in STRUCTURE and sNMF) and the others were sampled separately (Supplementary Table 1). Further, we concatenated 907 RAD loci for phylogenetic reconstruction for a more accurate tree topology (Leache et al., 2015) *via* the –phylip-var-all option in Stacks 2.2 (Catchen et al., 2013). BEAST analysis (Bouckaert et al., 2014) was conducted with empirical site frequency and GTR+GAMMA in the substitution model and the gamma distribution birth rate in the Yule model prior with other settings default (xml file in figshare).^1^ We performed five independent runs, each conducting 50 million Markov chain Monte Carlo (MCMC) simulations and sampling every 1,000 simulations. The convergence and the posterior probability were then checked by Tracer 1.7 (Rambaut et al., 2018). The independent runs were all converged, and we selected the one with the highest posterior probability and effective sample size (ESS) > 200 as the input for TreeAnnotator (Bouckaert et al., 2014). The target tree was summarized by maximum clade credibility with 40% burn-in.

### Tree-Based Species Delimitation by Discovery Methods

We used the individual tree to validate the species boundary using the discovery methods (Carstens et al., 2013) by the single-locus species delimitation, general mixed Yule-coalescent (GMYC) (Fujisawa and Barraclough, 2013), and Poisson tree processes (PTP) (Zhang et al., 2013), based on concatenated RAD loci (Fujisawa and Barraclough, 2013). We implemented the calibrated tree from BEAST2 in multiple threshold GMYC (mGMYC) and the Bayesian version of GMYC (bGMYC) in splits (Ezard et al., 2017) and bGMYC packages (Reid, 2014), respectively. In mGMYC, the target tree was estimated using the gmyc function. In bGMYC, 500 individual subtrees from BEAST were randomly sampled for 50,000 MCMC simulations, a 100 thinning interval, and 10% burn-in by the bgmyc.multiphylo function. The convergence was visualized by the spec.prombat function.

The PTP method considering only the number of substitutions was implemented in multi-rate PTP (mPTP) by standalone mptp (v 0.2.4) software (Kapli et al., 2017) and the Bayesian version of PTP (bPTP) in an online webserver.^2^. In mPTP, the minimum branch length threshold was detected by –minbr_auto and four independent runs with 10 million MCMC samplings; a 1,000 sampling interval and 10% burn-in were set. The combined likelihood plot and the three starting delimitations (i.e., randomly generated, maximum likelihood, and single exponential delimitation) were used to assess the convergence. For bPTP performed using the online webserver, the species were delimited by a target Bayesian individual tree with 500,000 generations with 100 sampling intervals and 20% burn-in. Both original maximum likelihood (PTP ML) and Bayesian implementations (bPTP) were reported.

### Morphometric Analyses

**FIGURE 4:**
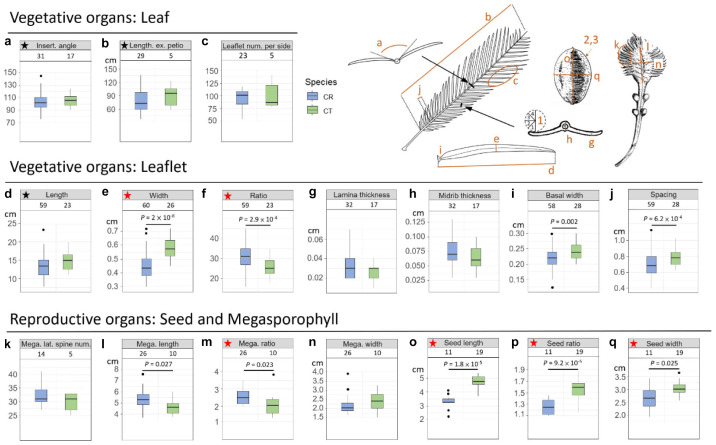
Morphometrics of leaf, leaflet, seed, and megasporophyll. The number block is the sample size. The ratio is calculated as the length divided by the width. The black and red stars are not significant and significant, respectively, diagnostic traits between *Cycas revoluta* and *Cycas taitungensis*. The draft of the measured organ is modified from Li and Keng (1975). Insert. angle, insertion angle; Leaflet num. per side, leaflet number per side; Mega. lateral spine num., megasporophyll lateral spine number; Mega. length, megasporophyll lamina length; Mega. ratio, megasporophyll lamina ratio; Mega. width, megasporophyll lamina width. The part labels **a–q** are the plant trait corresponding to depiction and Table 2.

In addition to genetic evidence, the morphological variations were also scrutinized to evaluate species status. Eleven vegetative and 10 reproductive diagnostic characters (Wang, 1996; De Laubenfels and Adema, 1998; Chen and Stevenson, 1999; Whitelock, 2002; Walters and Osborne, 2004; Hill, 2008) were selected for *Cycas* classification (Figure 4 and Table 2). Although the female cone compactness is a diagnostic trait between *C. revoluta* and *C. taitungensis*, we did not consider it because of the difficulty standardizing it due to large variation through the developmental stage (Walters and Osborne, 2004).

**TABLE 2:** Measurement of vegetative and reproductive traits in two species.

Organs	Traits	*n*	Mean	Min, Max	*n*	Mean	Min, Max
			
		*Cycas taitungensis*			*Cycas revoluta*		
Vegetative: Leaf	a. Insertion angle	17	105.75	90.56, 124.39	31	104.76	75.86, 146
	b. Length*	5	90.1	58.16, 122.51	29	78.21	37.18, 136
	c. Leaflet number*	5	101.4	76, 141	23	96.48	54, 118
Vegetative: Leaflet	d. Length	23	14.8	10.87, 19.89	59	13.37	7.72, 23.53
	**e. Width**	**26**	**0.58**	**0.45, 0.72**	**60**	**0.44**	**0.3, 0.72**
	**f. Ratio**	**23**	**25.77**	**18.12, 34.77**	**59**	**30.72**	**15.87, 44.82**
	g. Lamina thickness	17	0.028	0.01, 0.04	32	0.031	0.02, 0.07
	h. Midrib thickness	17	0.061	0.03, 0.1	32	0.073	0.03, 0.13
	**i. Basal width**	**28**	**0.24**	**0.2, 0.3**	**58**	**0.22**	**0.11, 0.3**
	**j. Spacing**	**28**	**0.79**	**0.62, 1.03**	**59**	**0.69**	**0.4, 1.13**
	1. Recurvation	20	−	−	168	−	−
Reproductive: Megasporophyll	k. Lateral spine number	5	29.8	25, 33	14	32.36	27, 41
	**l. Length**	**10**	**4.66**	**3.8, 6**	**26**	**5.39**	**3.7, 7.6**
	**m. Ratio**	**10**	**2.11**	**1.3, 3.87**	**26**	**2.54**	**1.93, 3.47**
	n. Width	10	2.38	1.52, 3.24	26	2.18	1.63, 3.93
Reproductive: Seed	**o. Length**	**19**	**4.73**	**3.58, 5.34**	**11**	**3.3**	**2.19, 4.14**
	**p. Ratio**	**19**	**1.56**	**1.15, 1.87**	**11**	**1.25**	**1.11, 1.45**
	**q. Width**	**19**	**3.06**	**2.56, 3.65**	**11**	**2.65**	**1.93, 3.45**
	2. Sclerotesta groove	6	−	−	5	−	−
	3. Sarcotesta color	19	−	−	11	−	−

*Leaf length is measured without petioles and the leaflet number is measured on one side. *Alphabet letter and number match Figure 4 and bold font indicates a significant difference between species. *Alphabet letter and number denote continuous and categorical traits, respectively.

The traits were measured in 109 specimens from nine herbaria (74 *C. revoluta* and 35 *C. taitungensis*) and 21 *C. taitungensis* from the coastal mountain range (*N* = 16) and the National Chung Hsing University campus (*N* = 5, cultivated individuals from Hongye Nature Reserve) (Supplementary Table 1). Specimen from herbaria HAST, KAG, KYO, PE, TAI, TAIF, TI, TNS, and UPS or their websites were examined. Herbarium acronyms follow Index Herbariorum (Thiers, 2019, continuously updated). Specimens with unclear collection information were excluded.

**FIGURE 5:**
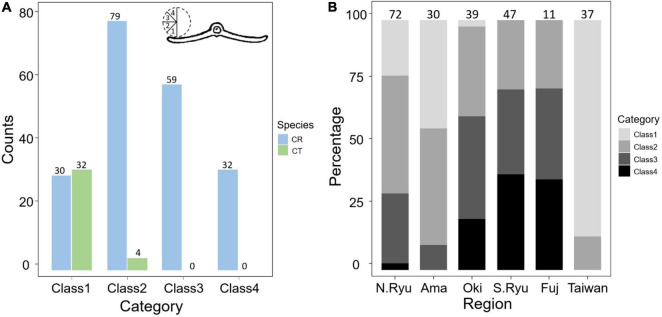
Comparisons of the revolute extent in the leaflet margin **(A)** between species and **(B)** among geological regions. The number above the bars are measured sample size. CR, *Cycas revoluta*; CT, *Cycas taitungensis*. The cross section of leaflet is modified from Li and Keng (1975).

To measure the recurvation of leaflet margin, an additional 188 samples of leaflets used for DNA extraction (178 *C. revoluta* and 10 *C. taitungensis*) were examined (Supplementary Table 1). The recurvation degree of the leaflet margin was categorized into four classes (Figure 5A). The middle part of dried mature leaflets was applied to maintain consistency in the measurements. The Mann–Whitney *U*-test was used for testing the significant differences of interspecific variations by wilcox.test function of the stats package. Finally, four significantly different interspecific traits relevant to leaflets (i.e., leaflet width, ratio, basal width, and spacing; see “Results”) were measured to construct the island dendrogram. The traits were scaled first and transformed by Euclidean distance using the dist function of the stats package. Among four clustering methods [unweighted pair group method with arithmetic mean (UPGMA), complete and single linkage, and Ward’s method], the best one was chosen by evaluating the agglomerative coefficient. Then the dendrogram was drawn by the agnes function in the cluster package (Maechler et al., 2012).

## Results

### Bioinformatics of ddRAD-Seq and Genetic Diversity

After demultiplexing and mapping 194,992,789 pair-end reads from 248 samples, the average contig size was 242.8 bp with a mean overlap size of 28.9 bp. Among 806,955 loci, the mean coverage was 59.8× (minimum = 28.3×). The population program kept 2,055 loci with a mean length of 238.21 bp and a total length of 491,971 bp. After quality control, there were 907 unlinked SNPs among 239 individuals (*n* = 209 and 30 for *C. revoluta* and *C. taitungensis*, respectively) with a missing rate of 35.4%. Among 110 selected individuals for individual phylogenetic reconstruction, there were 188,059 bp with 3,509 SNPs in 907 concatenated RAD loci. The genetic diversity showed fewer private alleles, but higher *He* and π in *C. taitungensis* (*He* = 0.238; π = 36.58; private allele = 108) than *C. revoluta* (*He* = 0.138; π = 27.35; private allele = 260). The species divergence was low (*F*st = 0.077). The SNP data were deposited in the Figshare repository and are available at https://doi.org/10.6084/m9.figshare.16987954.v1.

### Discordance of the Population Structure With Geographic Gaps and Larger Intraspecific Than Interspecific Genetic Divergence in *Cycas* Sect. *Asiorientales*

The island population structure revealed *K* = 4 and 7 as the best clustering in sNMF and StrAuto, respectively (Supplementary Figure 1). Both results separated two groups clearly, north of the Amami Islands (hereafter the northern group) and south of the Okinawa Islands (hereafter the southern group) in the middle of the central Ryukyus (Figure 2). The genetic structure did not correspond to the geographic gaps and the species boundary. The genetic compositions across the Tokara and Kerama Gaps were more homogeneous with red and orange genetic components (Figure 2). At the species level, the genetic compositions of *C. taitungensis* were similar to *C. revoluta* of the central and northern Ryukyus except for the green lineage. The inconsistency of the geographic boundary also appeared in the Fujian population with similar genetic compositions with the northern group, which was geographically separated from Fujian by the Okinawa Trough.

**FIGURE 6:**
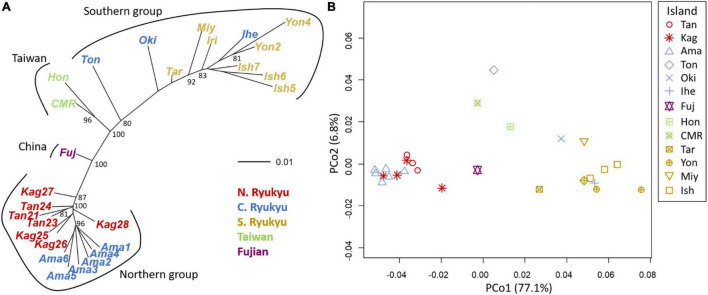
Population genetic structure inferred by **(A)** the neighbor-joining tree and **(B)** principal coordinate analysis (PCoA) by Nei’s distance. The island names are abbreviated as the first three characters of the full names, except for CMR (Coastal Mountain Range). Within-island populations are listed in Supplementary Table 1.

**FIGURE 7:**
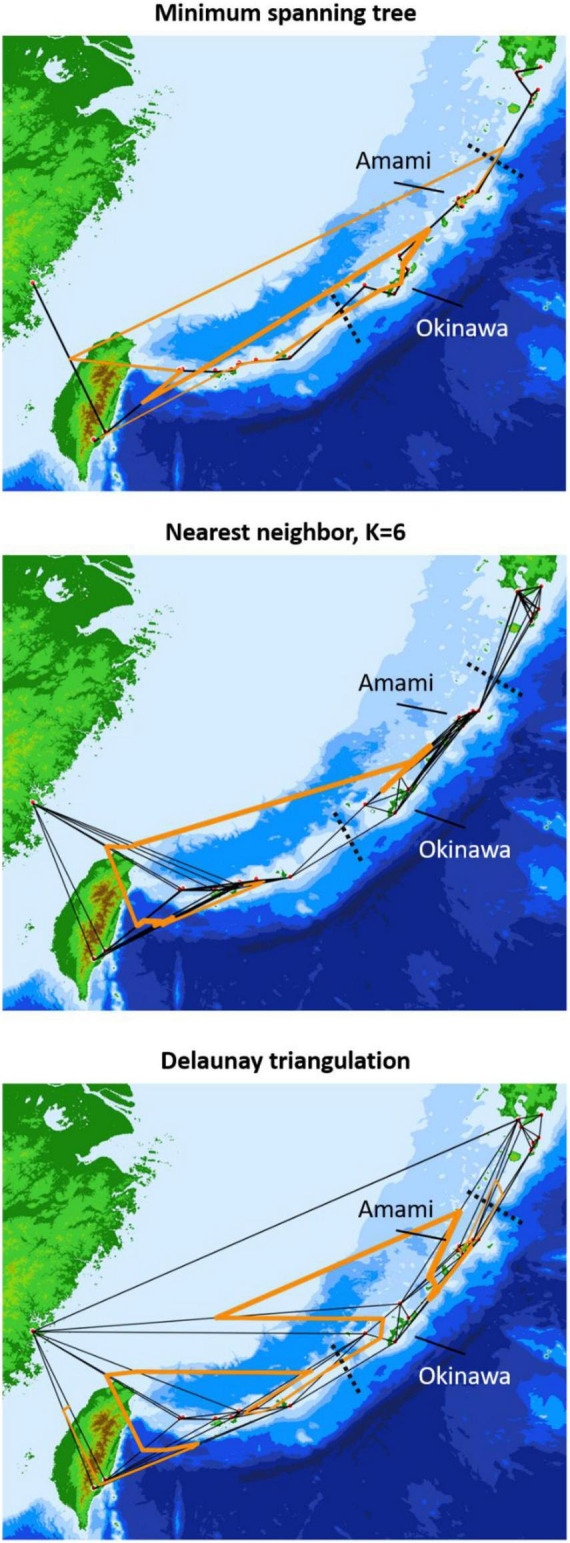
Population genetic boundaries based on three connection networks. The solid black and orange lines represent the connection network and barriers, respectively, and the dashed black lines are the geological boundaries separating the northern, central, and southern Ryukyus.

The neighbor-joining tree and PCoA consistently exhibited greater intraspecific divergence of the northern and southern groups of *C. revoluta* than the interspecific divergence (Figure 6). Although *C. taitungensis* formed a monophyletic group, both northern and southern groups of *C. revoluta* were more closely related to *C. taitungensis* than each other (Figure 6A). The population barriers and island pairwise *F*st also indicated stronger intraspecific than interspecific variations (Figure 7 and Supplementary Table 4). In three connection networks, the major barrier was congruently assigned as the strongest barrier between the southern and northern groups (pairwise *F*st: 0.32–0.61), rather than between *C. taitungensis* and *C. revoluta* in the Ryukyus (*F*st: 0.056–0.273) and between Fujian and the southern group (*F*st: 0.11–0.298). For the weaker interspecific barrier, most *C. taitungensis* individuals were genetically admixed with *C. revoluta*, except for six individuals harboring the nearly fixed green genetic component. Consequently, the interspecific barrier assigned by Nei’s distance was mainly ascribed to this distinct cryptic green lineage. Another minor barrier that appears in the Tokara Gap in the MS and DT connection networks was concordant with the different genetic compositions of the Amami Islands in the northern group in sNMF when *K* = 7 (Figure 2).

### Ongoing but Incomplete Speciation After the Divergence

Among the 100,000 simulations, 13 summary statistics were retained for the further model selections and parameter estimation after correlation removal. The confusion matrix in model validation discerned the four models appropriately, and the multidimensional space from summary statistics embraced the observed value in the PCA (Supplementary Figure 2). After model selection using 1 million simulations, the continuous gene flow was the best model with the highest Bayes factor and posterior probability (Table 3). Moreover, the observed data fit well with the simulated ones in the goodness-of-fit test (*P* = 0.51) (Supplementary Figure 3).

**TABLE 3:** Bayes factor and posterior probability of the approximate Bayesian computation models.

Model	Bayes factor	Posterior probability
**CG**	**39.14**	**0.53**
SC	30.10	0.4
CI	0.27	0.013
PC	3.69	0.05

*CG, continuous gene flow; SC, secondary contact; CI, complete isolation; PC, primary contact. The bold face indicated the best supported model based on Bayes factor and posterior probability.*

Regarding parameter estimation of the continuous gene flow model (Figure 3, Supplementary Figure 4, and Table 4), sect. *Asiorientales* diverged from its sister group t3 generations ago [weighted median: 162,546.4; weighted 95% highest posterior density (HPD): 26,558–282,427] with a large effective population size (weighted median: 37,409.2), and the two species diverged t2 generations ago (weighted median: 4,588.2; weighted 95% HPD: 51–86,158). At t1 generations ago (weighted median: 109.7; weighted 95% HPD: 15.5–4,176), bottleneck events occurred in both species with N_CR1_ (weighted median: 34,430.6) contracted 83% to N_CR2_ (weighted median: 5,835.7; weighted 95% HPD: 2,002.8–8,471.5), which is similar to a 77% contraction in N_CT1_ (weighted median: 12,067.5) to N_CT2_ (weighted median: 2,669.8; weighted 95% HPD: 614.2–4,697.1) that is observed today. Notably, the current larger effective population size of *C. revoluta* than *C. taitungensis* contradicts the findings of Chang et al. (2019). Following bottleneck events, the interspecific migration rates also decreased about an order of magnitude from m_CT1_ (weighted median: 3.1 × 10^–4^; weighted 95% HPD: 1.6 × 10^–7^–0.44) to m_CT2_ (weighted median: 1.8 × 10^–5^; weighted 95% HPD: 6.8 × 10^–8^–0.041) and m_CR1_ (weighted median: 1.4 × 10^–5^; weighted 95% HPD: 5.7 × 10^–8^–0.02) to m_CR2_ (weighted median: 4 × 10^–6^; weighted 95% HPD: 7 × 10^–8^–2.5 × 10^–4^). This reduced interspecific gene flow also indicates ongoing speciation. Nonetheless, the migration from *C. taitungensis* to *C. revoluta* is stronger than in the opposite direction.

### Northward Serial Divergence of *Cycas* Sect. *Asiorientales*

**FIGURE 8:**
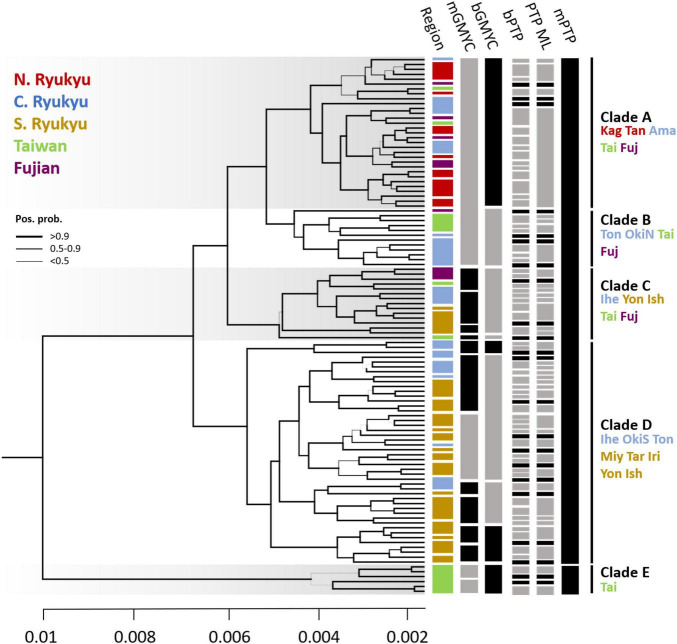
The Bayesian individual tree reconstructed by concatenated restriction site-associated DNA (RAD) loci and the species delimitation results. The branch width indicates the posterior probability. The color bar in each region corresponds to geological regions (i.e., N., C., and S. Ryukyu; Taiwan; and Fujian). For the species delimitation results, the gray bars are posterior probabilities < 0.6. The island names are abbreviated as the first three characters of the full names. OkiN and OkiS represent the northern and southern Okinawa populations, respectively. The bar is unscaled time with a substitution rate equal to 1.

The individual tree revealed polyphyly in *C. taitungensis* and *C. revoluta* and could be separated into five clades (A–E) with high posterior support (Figure 8). In terms of geography, there was a propensity of serial divergence in sect. *Asiorientales* from south to north of the Ryukyu Archipelago. Clade E representing the cryptic green lineage in *C. taitungensis* diverged first from other lineages. The subtending Clades C and D include all southern Ryukyus, plus the Iheya, Tonaki, and southern Okinawa Islands of the central Ryukyus. Specifically, Clade C was admixed with *C. taitungensis* and Fujian individuals, so Clades A and B diverged last. Considering Clades A and B, the former included Tonaki Island and the northern Okinawa Islands of the central Ryukyus, and the latter included the northern group.

### Inconsistency of Species Delimitation

The species delimitation failed between the two cycads. If *C. revoluta* and *C. taitungensis* are two “good” species, we expected a distinguished species boundary. However, the paraphyly of sect. *Asiorientales* rejected the delimitation of *C. revoluta* and *C. taitungensis* based on tree-based delimitation approaches (i.e., GMYC and PTP analogs). Results of GMYC and PTP reflect the intraspecies level (Figure 8). For GMYC, the multi-threshold and Bayesian versions obtained similar patterns. In mGMYC, 14 groups were recognized; Clades A and B clustered together, while four, seven, and two groups were recognized in Clades C, D, and E, respectively. bGMYC detected only nine groups that recovered Clades A, B, and E, while separating two and four groups in Clades C and D, respectively.

For PTP, the results were incongruent with GMYC and between multi-rate (mPTP) and non-multi-rate PTP versions (PTP ML and bPTP) (Figure 8). The Bayesian and likelihood versions were oversplit, with 81 and 60 groups discovered, respectively. The nearly individual-level separation indicated a strong false positive of delimitation (Luo et al., 2018). The mPTP outperformed the former version by accounting for intraspecific variation with different exponential distributions. As expected by Kapli et al. (2017), the mPTP yielded fewer putative species than PTP with Clade E recovered and merging of Clades A–D.

### Overlapped Morphological Variation Between *Cycas taitungensis* and *Cycas revoluta*

Among 11 vegetative and 10 reproductive traits, there were nine traits (four vegetative and five reproductive) significantly different between the species (Table 2), and six of them were the described diagnostic traits (red stars in Figure 4 and leaflet recurvation in Figure 5). In general, *C. taitungensis* yielded wider and longer leaflets with larger inter-leaflet spacing and a wider basal width than *C. revoluta*, and the megasporophyll tended to be ovate and shorter with larger and longer seeds. However, these variations were continuous with *C. taitungensis* included in *C. revoluta*.

For the five non-significant reproductive traits, counterexamples were found between the two species for three diagnostic traits, namely, seed shape, sarcotesta color, and sclerotesta groove Supplementary Figures 5–7). The indistinguishable sclerotesta groove affirmed the descriptions from Hill (2008). For the seven non-significant vegetative traits, four diagnostic traits (leaflet recurvation, leaflet length, leaf insertion angle, and leaf length) were not different between the two species (black stars in Figure 4). Even though the epithet of *C. revoluta* is derived from the recurved leaflet margin, 15% of leaflet margins were slightly or not recurved (class 1) and, hence, were not discernible from *C. taitungensis*. Most flattened leaflets belonged to the northern group (Figure 5). Alternatively, the typical recurved leaflet margins were predominantly observed in the Fujian and southern groups. Focusing on the Ryukyus and Kyushu, these patterns are consistent with our and previous population genetic clustering (i.e., the northern and southern groups) (Kyoda and Setoguchi, 2010), pairwise *F*st, and leaflet morphometric cladogram (Figures 2, 6, Supplementary Figure 8, and Supplementary Table 4; see also Setoguchi et al. (2009)).

Among four clustering methods, Ward’s method explained the most structure (agglomerative coefficient 0.94). The compound traits integrating the four significantly different leaflet traits failed to distinguish the species and showed the disjunct distribution of phenotypically similar populations. The dendrogram revealed a similar topology to the Bayesian individual tree, in which *C. taitungensis* was primarily clustered with the Fujian, Okinawa, and northern groups (Supplementary Figure 8). In summary, half of the diagnostic characters (5/10) were significantly different between species, but the variation of all interspecific traits overlapped.

## Discussion

Cycads are conserved in morphology and genetics. Comprehensive morpho-genetic data help validate taxonomic status by tracking their speciation history. This study combined the genome-wide ddRAD and morphological traits to illuminate the evolutionary history and reappraise species status of sect. *Asiorientales*. The ABC results indicated non-allopatric divergence following continuous gene flow. The morpho-genetic evidence unveiled larger intra- than inter-species variations laid the foundations of species reappraisal in sect. *Asiorientales*.

### Continuous but Decreasing Gene Flow Supports Recent Non-allopatric Speciation

Our best ABC model suggests a continuous gene flow with the bottleneck after the divergence between *C. revoluta* and *C. taitungensis*. This scenario indicates non-allopatric speciation. The origin of sect. *Asiorientales* around 4.8 mya, as calculated as 30 × t3 (162,546 generations, Figure 3 and Table 4), matches the timing of the emergence of the Ryukyu Arc as a series of continental islands ca. 5–10 mya (Wang et al., 2014; Government of Japan, 2019), underpinning the vicariance of sect. *Asiorientales* from its sister sect. *Panzhihuaenses* on the continent (Xiao and Moller, 2015; Mankga et al., 2020). Incipient speciation occurred in the middle Pleistocene (t2, ca. 0.14 mya) after the synchronous isolation of the Ryukyu Arc into the northern, central, and southern Ryukyus, and Taiwan around 1.55 mya (Osozawa et al., 2013). Since then, isolation between the southern Ryukyus and Taiwan may have persisted due to the Yonaguni Strait (Gallagher et al., 2015; Zheng et al., 2016; Government of Japan, 2019). Nevertheless, the gene flow was continuous, thus rejecting allopatric speciation. The mismatch between the speciation event and the simultaneous landscape fragmentation suggests that continuous gene flow delayed the speciation, prolonged the lineage sorting time, and preserved high ancestral polymorphisms. Consequently, current spatial morphogenetic variations are shaped by both gene flow and ancestral polymorphism.

**TABLE 4:** Parameter estimation of the approximate Bayesian computation continuous gene flow model.

Parameter	Weighted mean	Weighted median	Weighted 95% interval
N_CR2_	5590.3	5835.7	2002.8–8471.5
N_CT2_	2607.7	2669.8	614.2–4697.1
N_anc_REL_	3	3.1	0.55–5.42
N_CR_REL_	5.7	5.9	1.4–9.4
N_CT_REL_	4.3	4.52	1–7.02
t1	703.1	109.7	15.5–4175.9
t2	27,840	4588.2	51.1–86158.1
t3	160505.4	162546.4	26558.1–282427.4
m_CT1_	1.2E-1	3.1E-4	1.6E-7–4.4E-1
m_CR1_	2.2E-3	1.4E-5	5.7E-8–2E-2
m_CT2_	3.5E-3	1.8E-5	6.8E-8–4.1E-2
m_CR2_	1.5E-4	4E-6	7E-8–2.5E-4
μ	9.9E-8	9.87E-8	1.02E-8–2E-7

*The parameters are defined in Table 1.*

#### Mechanisms of Inter-Island Gene Flow After Divergence

After speciation, inter-island gene flow was facilitated by sea-level change. The land bridges prevailed in the Last Glacial Maximum (LGM) among the Ryukyu Arc, facilitating the colonization of species with poor transoceanic dispersal ability (Government of Japan, 2019). Transoceanic dispersal of cycad seeds and pollens has been documented. Cycad’s seed dispersibility varies from less than 1 meter to transcontinental by both abiotic (e.g., gravity, sea current, and rain) and biotic media (Burbidge and Whelan, 1982; Hall and Walter, 2013; Cousins and Witkowski, 2017; Liu et al., 2021a). However, some species could not be transported by sea due to a lack of spongy layer in seed, such as sect. *Asiorientales* (Dehgan and Yuen, 1983). Therefore, the stepping stone process was supposed in inter-island colonization of sect. *Asiorientales via* land bridge in LGM. The northward colonization may be dominant based on the calibrated individual tree (Figure 8). The genetic compositions remained homogeneous across the long-lasting Kerama and Tokara geological barriers than the Amami-Okinawa genetic boundary (Ota, 1998), indicating that further ecological factors might engage in the divergence of northern and southern groups.

In addition to stepping stone colonization, overseas gene flow may occur between the two more distantly distributed species after the bottleneck event. The overseas gene flow would be mainly from pollen carried by wind and insects. However, the wind-pollinated distance is short in cycads, and hence insects would be more likely as overseas pollination medium (Kono and Tobe, 2007). The primary pollinator, *Carpophilus chalybeus*, is broadly distributed in Taiwan and the Ryukyu Archipelago (Kirejtshuk, 2005; Chung and Shao, 2021). With the overlapped coning period between *C. revoluta* and *C. taitungensis* in spring to summer, insect pollination would facilitate bidirectional gene flow when the northeast and southwest monsoon are prevalent. Northward moving typhoons may also cause more frequent northward gene flow.

According to the individual tree, the gene flow would be spatially heterogeneous, suggesting other potential mechanisms (Figure 8). Apart from Clade E, all *C. taitungensis* individuals were closely related to Clades A–C of *C. revoluta*, which may be caused by more frequent gene flow than between *C. taitungensis* and Clade D of *C. revoluta* (Fontaine et al., 2015). Although the transoceanic seed dispersal of sect. *Asiorientales* is limited (Dehgan and Yuen, 1983; Liu et al., 2021a), other diaspores, such as bulbils, may lead to spatially heterogeneous gene flow. Many land plant transoceanic dispersal cases only focused on seeds, but few on vegetative dispersals (Ridley, 1930; Vargas et al., 2014, 2015; Price and Wagner, 2018). Bulbils are the vectors for fast and genetically homogeneous population colonization (Norstog and Nicholls, 1997; Thomas et al., 2005; Mizuki et al., 2010; Huang and Hsieh, 2014). Successful and profuse vegetative propagation has been reported in many cycad species (Raju and Jonathan, 2010; Raju and Rao, 2011; Cousins and Witkowski, 2017), but the importance of its transoceanic dispersal is less discussed (Ridley, 1930; Keppel, 2001). Bulbil development is commonly observed in near-sea streamside *C. taitungensis* and seashore *C. revoluta* (Stopes, 1910; Stevenson, 2020) and would lead to long-distance dispersal by hitchhiking by the Kuroshio Current, although not formally recorded. The Kuroshio Current is strong and warm, persistently flowing northwestward from the eastern Philippines, offshore eastern Taiwan, and along the western Ryukyu Archipelago. It changes its course eastward to the Pacific Ocean at the Tokara Gap (Gallagher et al., 2015; Figure 1). This route could be dated back to ca. 2–1.5 mya after the speciation of *C. revoluta* and *C. taitungensis*. The likelihood of the Kuroshio promoting dispersal and landing on islands is low but possible, especially under monsoon support (Kurita and Hikida, 2014; Yang et al., 2018; Kaifu et al., 2020). Over a period of 30 years, Kaifu et al. (2020) recorded that ca. 7% of drifters (8/114) analogous to bulbils reached the northern Ryukyus with higher probability than the central or southern Ryukyus by the Kuroshio, set off from Taiwan within half a month (for the coral species example, see Selmoni et al. (2020)), faster than the adventitious root development (ca. 1 year) in cycad bulbils (Dehgan, 1997; Stevenson, 2020).

The occasional long-distance vegetative dispersal following its faster growth and colonization (compared with seedlings) facilitates a founder-takes-all situation (i.e., new colonizers on a new habitat expand rapidly and block the latecomers; Waters et al., 2013). This spatially heterogeneous gene flow and genetic influence may explain the disjunct distribution and barrier patterns of morphogenetically similar populations of *C. taitungensis* in Taiwan and *C. revoluta* in the central and northern Ryukyus (Figure 7). The population genetic structure, pairwise *F*_ST_, and paraphyletic individual tree demonstrated the cohesiveness of *C. taitungensis* and the central and northern Ryukyus of *C. revoluta* and not the southern Ryukyus (Figures 2, 6, 8 and Supplementary Table 4), similarly to the morphology, which exhibits less recurvation, a larger length-to-width ratio, spacing, and a wider leaflet and basal width (Figures 4, 5). Specifically, the typical form of strongly recurved leaflets in *C. revoluta* was discovered mainly in the southern group. These similarities of disjunct morphogenetic variations correspond to leapfrog patterns, which underlie the disjunct distribution of phenotypically similar populations (Remsen, 1984).

#### Causes of the Leapfrog Morphogenetic Pattern

There are two main hypotheses for the leapfrog pattern (Remsen, 1984; Cadena et al., 2011; Marquez et al., 2020). The first hypothesis is the shared common ancestor of disjunct populations, which could be attributed to the long-distance Kuroshio hitchhike of bulbils. The spatially heterogeneous gene flow decelerates the lineage sorting and retains a more common ancestral polymorphism between *C. taitungensis* and the northern group of *C. revoluta*. The second hypothesis is the evolutionary convergence of leapfrogged populations or adaptive divergence of the intervening populations, which this study cannot validate. The convergence would occur between *C. taitungensis* and the northern group of *C. revoluta.* Multiple selective sweeps may contribute to adaptive divergence of the southern group of the *C. revoluta.* Along the Ryukyu Archipelago, the leapfrog pattern has been discovered in other species (Matsumura et al., 2009), but has not been tested to be driven by natural selection or stochastic process. Consequently, future studies can assess possible environmental and geographic forces influencing the evolution of functional genes to test these hypotheses.

### The Species-Definition Anomaly Zone Leads to Problematic Taxonomy

The phenomenon of the greater intraspecific divergence than between species is called the species-definition anomaly zone. It can occur when two species harbor extremely different geographical distributions under gene flow. The subdivided population will inflate the effective population size for the species with a larger range and facilitate the species-definition anomaly zone (Wang and Caballero, 1999; Jiao and Yang, 2021). The effective population size of *C. revoluta* is two times that of *C. taitungensis*, in concert with the spatially heterogeneous gene flow, possibly enhancing the impact of the species-definition anomaly zone. The larger intra- than inter-specific variations indicate the problematic taxonomy for *C. taitungensis* and *C. revoluta*. The morphogenetic leapfrog pattern suggests that the divergence of *C. revoluta* and *C. taitungensis* should be regarded at the population level rather than the species level, considering them as separately evolving metapopulations (based on the unified species concept) (Wiley, 1978; De Queiroz, 2007).

Reduced gene flow and allopatric distribution may have initiated the speciation of these two cycads. However, recent human-induced sympatry by the horticultural introduction of *C. revoluta* into Taiwan may disrupt *C. taitungensis* speciation (Chen and Stevenson, 1999) by messing with the gene pool of natural populations (Chiang et al., 2013). The compromise between natural and anthropogenic processes is worth considering in our case of *C. revoluta* and *C. taitungensis*.

### Taxonomy Status of *Cycas taitungensis*: A Synonym of *Cycas revoluta*

#### Incomprehensive Morphological Comparisons Misclassify the Interspecific Variation

The leapfrog morphogenetic variations and compatible reproduction under the same coning time indicate interconnected evolutionary fates. Our previous model-based analyses also support high ancestral polymorphism with recent gene flow (Chiang et al., 2009, 2018). Genetic studies have consistently demonstrated the cohesion of the two species, but morphological studies are more complicated, especially regarding species diagnosis and morphological comparisons.

The misidentification of the two species is not only because *C. taitungensis* had long been misidentified as *C. taiwaniana*, but also because of the problematic morphological comparisons in the *C. taitungensis* protolog. The earliest collection of *C. taitungensis* was made by Shun-ichi Sasaki in 1920. His collection was identified as *C. taiwaniana* native to the continent (southern China) and classified in sect. *Stangerioides* until Shen et al. (1994) published *C. taitungensis* as a new species. However, its morphological comparisons are questionable due to the cited reference (Yamamoto, 1928), which erroneously compared *C. revoluta* with mixed samples of genuine *C. taiwaniana* and *C. taitungensis*. Yamamoto (1928) described that *C. taiwaniana* has longer leaflets, leaves, and petioles, a smaller revolute margin, a larger insertion angle, and more leaflets. For the reproductive organs, the shape of the male cone is subcylindrical, and the megasporophyll is orbicular to ovate for *C. taiwaniana* with glabrous ovule (oblong or ovate to lanceolate with tomentose ovule for *C. revoluta*). However, tomentose ovule is a synapomorphy of sect. *Asiorientales* (Walters and Osborne, 2004), and Yamamoto’s description suggest that the cited specimens had been misidentified. This also implies that the taxonomic studies (Kanehira, 1936; Li and Keng, 1954, 1975; Liu, 1960) following Yamamoto’s work are questionable (Shen et al., 1994).

After the publication of *C. taitungensis* (Shen et al., 1994), some questionable examined specimens were still from unknown or erroneous distributions (Chen and Stevenson, 1999; Whitelock, 2002). In addition, due to large morphological variations, insufficient specimen measurements also lead to biased diagnoses. Wang (1996) and Hill (2008) first compared specimens correctly, but their comparison may be biased regionally. They distinguished *C. taitungensis* from *C. revoluta* based on (1) longer and flatter non-keeled leaf and non-revolute leaflet, (2) darker and larger seeds, and (3) more tightly, cabbage-like female cones. However, Wang (1996) and Hill (2008) only examined *C. revoluta* from Fujian and one individual from the northern Ryukyus, respectively (Zheng et al., 2017). The bias also appeared in the seed diagnosis. Whitelock (2002) and Hill (2008) illustrated grooved or non-grooved sclerotesta of *C. revoluta* and *C. taitungensis*, contradicting other references that described *C. revoluta* and *C. taitungensis* as having a smooth and grooved sclerotesta, respectively (De Laubenfels and Adema, 1998; Chen and Stevenson, 1999). In summary, the aforementioned diagnostic characters were insufficient and did not consider all variations. Our study for a more comprehensive comparison revealed continuous variations in these traits.

#### Why Not Define Subspecies?

In many species, an allopatric or edge population like *C. taitungensis* of sect. *Asiorientales* in Taiwan with slight morphogenetic differences would be treated as subspecies. However, the subspecies treatment is not recommended in cycads (Walters and Osborne, 2004) and would also not be appropriate in our case. Subspecies are traditionally geographic variants of polymorphic species that may be in the early stage of speciation (Mayr, 1982). The applications, however, are not standardized, and there are pronounced differences across taxonomic groups (Table 1; Huang and Knowles, 2016). The incongruence derives from anchoring the discrete subspecies through the initial stage of a speciation continuum.

Statistically evaluating and comparing the divergence level from multidimensional axes between species and subspecies would be a more objective way of assigning ranks (Huang and Knowles, 2016). Species units may still be the greatest challenge for cycad systematics due to the lack of intraspecific data (Walters and Osborne, 2004), let alone comparing species and subspecies levels across the speciation continuum. Therefore, the level below species is not recommended. Even taking the broad view of subspecies (de Queiroz, 2020), equating it at the species level but within a more inclusive lineage, the larger intraspecific divergence between the southern and northern groups of *C. revoluta* than the interspecific divergence between *C. revoluta* and *C. taitungensis* also markedly reduces inclusiveness. Alternatively, if the three subspecies were assigned (i.e., Taiwan and the northern and southern groups of the Ryukyu Archipelago), the taxonomic units are still confused and oversplit due to several morphological variants on small islands (Satake, 1981; Whitelock, 2002) and based on our species delimitation results (Figure 8). In summary, synonymization would be the most appropriate taxonomic treatment.

### Taxonomic Treatment

According to the results and discussion, we established the following treatment.

#### *Cycas revoluta* Thunb. in Verhandelingen Uitgegeeven Door De Hollandse Maatschappy Der Weetenschappen, Te Harlem 20 (2): 424, 426–427 (1782)

= *C. taitungensis* C.F. Shen, K.D. Hill, C.H. Tsou and C.J. Chen in *Botanical Bulletin of Academia Sinica* 35:133–140 (1994). Type: Taiwan. Taitung county, growing on the steep mountain slope in the secondary forest or well-exposed areas. C. H. Tsou 825 (Holotype: HAST!, Isotype: A, BM, IBSC, K, NSW, NY, P, PE!, TAI!) syn. nov.

#### Distribution

*Cycas revoluta* is widely distributed along southern Kagoshima, the Ryukyu Archipelago, and eastern Taiwan. The distribution of natural populations in Fujian needs further verification (Zheng et al., 2017). In Japan, the habitats are mostly near the coastal area on the coral reef. Some populations are under or near forests with an altitude from sea level to 500 m. In Taiwan, two populations are located in the Taitung Hongye Village Taitung Cycas Nature Reserve (台東紅葉村台東蘇鐵自然保留區) along the Luyeh River Valley and the Coastal Range Taitung Cycas Forest Reserve (海岸山脈台東蘇鐵自然保護區), the western and eastern side of the East Rift Valley, respectively. Both populations generally grow on steep and exposed slopes with some in sparse forests at 300–900 m.

#### Conservation Implications

The threats and drivers of global biodiversity decline are mostly anthropogenically mediated (Mankga and Yessoufou, 2017), as is sect. *Asiorientales*. Extensive habitat disturbance and individual poaching have led to population fragmentation (e.g., *C. taitungensis* in Taiwan) and even possibly local extinction (e.g., *C. revoluta* in the Fujian population) (Shen et al., 1994; Chen and Stevenson, 1999; Whitelock, 2002; Huang et al., 2004). Secondary succession in lowland forest and pests, such as cycad aulacaspis scale (CAS) *Aulacaspis yasumatsui* and the cycad blue butterfly *Chilades pandava*, also reduce the habitat quality and individual health (Bailey and Lai, 2006; Wu et al., 2010). However, these threats are more severe for *C. taitungensis* than *C. revoluta*. The latest conservation assessment of the IUCN Red List revealed the improvement of *C. revoluta* from near threatened (NT) to least concern (LC) (Hill, 2010), while *C. taitungensis* has gone from vulnerable (VU) to endangered (EN) (Haynes, 2010). The worse conservation status of *C. taitungensis* is due to the increasing CAS and decreasing habitat quality and range size (Haynes, 2010). The genetic drift will enhance the subsequent reduction of heterozygosity if habitat loss continuous. With fewer private alleles than *C. revoluta* (108 vs. 260), the conservation status of the Taiwanese population will be inferior.

With the break of the species boundary, even if the Taiwan population is described as a unique evolutionary significant unit (ESU) (Chang et al., 2019), it will not be considered in the IUCN global assessment of species extinction risk. This situation signifies the negative effect of synonymized treatment on conservation evaluation. The absence of the conservation concern after synonym treatment has also appeared in other “globally common, but locally rare” taxa (Thakur et al., 2018). Under the considerations of evolutionary processes in conservation biology (Crandall et al., 2000), species as the only management unit is inappropriate (Casetta et al., 2019). Consequently, IUCN global assessment only at the species level would be insufficient.

Nevertheless, the guidelines for regional or national levels have also been proposed and could alleviate this demerit (IUCN, 2012). Such an index has been followed by the Taiwanese government to assess the conservation status of *C. taitungensis* and was classified as the “nationally critically endangered (NCR)” for its small area of occupancy (AOO) and fluctuation in the number of reproducible individuals (Yang et al., 2017). After our taxonomic treatment, the conservation of *C. revoluta* in Taiwan still needs to be considered on a national scale. This index could solve the predicament by considering the edge, higher evolvability, or regionally endangered populations and evaluating the conservation status more carefully. More attention on ESU at the regional or national scale would make the conservation management more comprehensive and focused.

#### Specimen Examined

The examined specimens from morphometrics are listed in Supplementary Table 5.

## Data Availability Statement

The datasets presented in this study can be found in online repositories. The names of the repository/repositories and accession number(s) can be found below: https://figshare.com/, https://doi.org/10.6084/m9.figshare.16987990.v1, https://figshare.com/, https://doi.org/10.6084/m9.figshare.16987954.v1.

## Author Contributions

P-CL conceived, designed the project, and interpreted the data with J-TC. J-TC collected field samples, performed lab experiments and statistical analyses, and wrote the manuscript. C-TC, KN, M-XL, and H-LL critically reviewed and provided constructive suggestions on the first draft. All authors read and approved the final manuscript.

## Conflict of Interest

The authors declare that the research was conducted in the absence of any commercial or financial relationships that could be construed as a potential conflict of interest.

## Publisher’s Note

All claims expressed in this article are solely those of the authors and do not necessarily represent those of their affiliated organizations, or those of the publisher, the editors and the reviewers. Any product that may be evaluated in this article, or claim that may be made by its manufacturer, is not guaranteed or endorsed by the publisher.

## Footnotes

1
https://doi.org/10.6084/m9.figshare.16987990.v1

2
http://species.h-its.org/ptp/
