# Corrigendum: Divergence with gene flow and contrasting population size blur the species boundary in *Cycas* Sect. *Asiorientales*, as inferred from morphology and RAD-seq data

**DOI:** 10.3389/fpls.2022.1081728

**Published:** 2023-01-12

**Authors:** Jui-Tse Chang, Chien-Ti Chao, Koh Nakamura, Hsiao-Lei Liu, Min-Xin Luo, Pei-Chun Liao

**Affiliations:** ^1^ School of Life Science, National Taiwan Normal University, Taipei, Taiwan; ^2^ Botanic Garden, Field Science Center for Northern Biosphere, Hokkaido University, Sapporo, Japan; ^3^ Department of Anthropology, Smithsonian Institution, National Museum of Natural History, Washington, DC, United States

**Keywords:** continental island, *Cycas*, Kuroshio, long distance dispersal, speciation, species concept

In the published article, there was an error in **Figure 4** as published. The draft of measured leaf trait was modified from journal which figure usage was not CC-BY. Therefore, the original modification was not allowed without permission, and the new figure should be provided.

There was also an error in the caption for **Figure 4**. The updated draft did not modify the figure from Setoguchi et al., 2009. We have corrected **Figure 4** and its caption.

**Figure 4:**
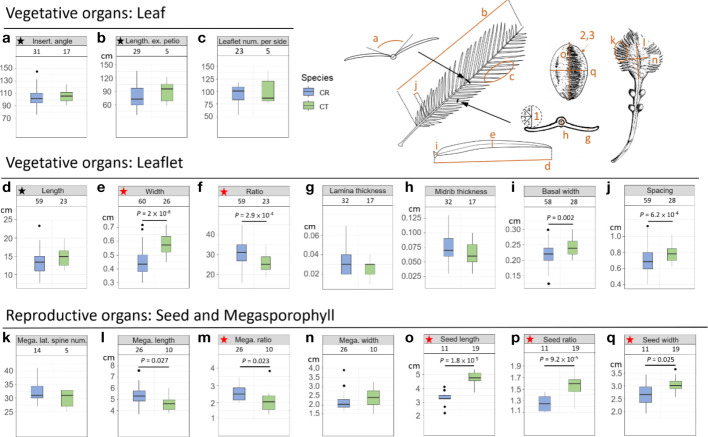
Morphometrics of leaf, leaflet, seed, and megasporophyll. The number block is the sample size. The ratio is calculated as the length divided by the width. The black and red stars are not significant and significant, respectively, diagnostic traits between *Cycas revoluta* and *Cycas taitungensis*. The draft of the measured organ is modified from Li and Keng (1975). Insert. angle, insertion angle; Leaflet num. per side, leaflet number per side; Mega. lateral spine num., megasporophyll lateral spine number; Mega. length, megasporophyll lamina length; Mega. ratio, megasporophyll lamina ratio; Mega. width, megasporophyll lamina width. The part labels a-q are the plant trait corresponding to depiction and Table 2.

The authors apologize for these errors and state that this does not change the scientific conclusions of the article in any way. The original article has been updated.

