# Supplementary material for: Divergence With Gene Flow and Contrasting Population Size Blur the Species Boundary in Cycas Sect. Asiorientales, as Inferred From Morphology and RAD-Seq Data
Source: Front Plant Sci. 2022 May 9;13:824158. doi: 10.3389/fpls.2022.824158 (PMC9125193; doi:10.3389/fpls.2022.824158)
Supplement: Supplementary file 1 [file Data_Sheet_1.docx]

**Divergence with gene flow and contrasting population size blur the species boundary in *Cycas* sect. *Asiorientales* from morphology and RAD-seq data**

**Running title: Blurred species boundary of *Cycas***

Jui-Tse Chang^1^, Chien-Ti Chao^1^, Koh Nakamura^2^, Hsiao-Lei Liu^3^, Min-Xin Luo^1^, Pei-Chun Liao^1,^*

1. School of Life Science, National Taiwan Normal University, Taipei 11677, Taiwan

2. Botanic Garden, Field Science Center for Northern Biosphere, Hokkaido University, Sapporo 060-0003, Japan

3. Department of Anthropology, Smithsonian Institution, National Museum of Natural History, Washington, DC, USA

* Corresponding author: Dr. Pei-Chun Liao

Tel: +886-2-77346330

Fax: +886-2-29312904

Email: [pcliao@ntnu.edu.tw](mailto:pcliao@ntnu.edu.tw)

Email addresses of authors:

Jui-Tse Chang: [b03612004@ntu.edu.tw](mailto:b03612004@ntu.edu.tw)

Chien-Ti Chao: [ff8bahamut@gmail.com](mailto:ff8bahamut@gmail.com)

Koh Nakamura: [kohnakamur@gmail.com](about:blank)

Hsiao-Lei Liu: [liuh@si.edu](mailto:liuh@si.edu)

Min-Xin Luo: [xji6aup3vup@gmail.com](mailto:xji6aup3vup@gmail.com)

ORIGINAL ARTICLE

**Method S1**

For the parameters setting of four models, effective population size (*Ne*), migration rate (m), and their time (t) in concert with mutation rate (µ) were estimated. In terms of the census population size (*Nc*), there were at least 1,744 and 3,000 individuals in *C. taitungensis* in Hongye of Taitung Province and *C. revoluta* in Toi Cape of southern Kyushu (from Taiwan forest bureau and Forestry and Fisheries Technical Information Society, Japan). The effective and census population size ratio (*Ne*/*Nc*) range from 0.05 to 0.2 (Frankham, 1995), and will skew to a smaller value for fluctuated population size, sex divisions, and high fecundity species such as cycads (effective population size and patterns of molecular evolution) (Charlesworth, 2009; Frankham et al., 2014). Therefore, at least 87.2 and 150 Ne were for *C. taitungensis* and *C. revoluta*, respectively. Although the IUCN red list estimated 10,000 and 100,000 – 200,000 of *Nc* for *C taitungensis* and *C. revoluta*, respectively, these values probably were overestimated (Zheng et al., 2017) and could be considered maxima value. Overall, the *Ne* of the two species was set to range from 50 – 5,000. After the demographic change, the relative population size ranged from 0.1 to 10, denoting sudden expansion and bottleneck, respectively. The migration rate referring to the probability of any gene migrating per generation ranged from 1×10^-9^ to 0.1. We take 30 years as generation time for the three-time parameters: time of sect. *Asiorientales* origin (t3), speciation time of *C. revoluta* and *C. taitungensis* (t2), and time of demography/migration change (t1). The upper bound of these three time were set to be 9 Ma (3×10^5^ generation), when the origin of *Cycas* was estimated (Nagalingum et al., 2011). The minima of t2 and t1was set around the first discovery of *C. revoluta* in 1782 (10 generations) and *C. taitungensis* in 1928 (5 generations), respectively. The mutation rate was set around the somatic substitution rate of *Picea* (2.7×10^-8^ site/generation) and neutral substitution rate of Cycadales (6.7×10^-10^ site/year) range from 2×10^-7^- 2×10^-9^ (site/generation) (De La Torre et al., 2017; Hanlon et al., 2019). Because of less weighting on lower order, the prior covering many orders of magnitude such as time and migration rate were set as logarithmic uniform distribution (Wegmann et al., 2009). However, we keep t3 in uniform distribution for the reasonably longer section origin time, and hence no need for weight adjustment on lower-order by log. The uniform distribution was also used in the rest of the parameters (*Ne*, relative *Ne*, and mutation rate).

After 1 million simulations, 5 thousand simulations were used for collinearity removal and model choice validation. The highly correlated summary statistics were removed with Pearson correlation |r|>0.9. Subsequently, the retained summary statistics in simulated and observed data were PCA transformed to confirm the model fits, then were input for model cross-validation in ABCtoolbox to search for the optimal summary statistics combinations for model choice. Ten simulations were used to reconstruct the confusion matrix in cv4postpr function in abc package by multinomial logistic regression with 0.05 tolerance.

After model selection through ABCtoolbox by best 1,000 simulations among 1million simulations, the goodness-of-fit was performed by gfit function with 100 replicates in abc package to evaluate the model fit against observed data.

**Table S1** Sampling and analyzed individuals information

| **Species** | **Biogeographic region** | **Locality** | **Sampled Population** | **N for ddRAD** | **N for BEAST2** | **N for leaflet revolution** | **Latitude** | **Longitude** |
| --- | --- | --- | --- | --- | --- | --- | --- | --- |
| *C. revoluta* | Eurasian mainland | Fujian | Fuj | 10 | 9 | 10 | 26.40246 | 119.65202 |
|  | Northern Ryukyu | Kagoshima | Kag 25 | 8 | 3 | 11 | 31.18401 | 130.62131 |
|  |  |  | Kag 26 | 8 | 4 | 8 | 31.15727 | 130.58757 |
|  |  |  | Kag 27 | 12 | - | 13 | 31.28219 | 131.12946 |
|  |  |  | Kag 28 | 10 | 1 | 10 | 31.00976 | 130.67226 |
|  |  | Tanegashima | Tan 21 | 10 | 3 | 6 | 30.49706 | 130.90034 |
|  |  |  | Tan 23 | 9 | 5 | 5 | 30.65128 | 130.94291 |
|  |  |  | Tan 24 | 9 | - | 5 | 30.76767 | 131.07006 |
|  | Central Ryukyu | Amami | Ama 1 | 8 | 4 | 4 | 28.47326 | 129.71536 |
|  |  |  | Ama 2 | 5 | 1 | 5 | 28.39971 | 129.50783 |
|  |  |  | Ama 3 | 6 | - | - | 28.46771 | 129.58913 |
|  |  |  | Ama 4 | 6 | 1 | 8 | 28.32081 | 129.27543 |
|  |  |  | Ama 5 | 5 | - | 6 | 28.21945 | 129.23744 |
|  |  |  | Ama 6 | 7 | 2 | 4 | 28.13215 | 129.36167 |
|  |  |  | Ama 7 | - | - | 4 | 28.49375 | 129.65866 |
|  |  | Iheya | Ihe | 10 | 8 | 6 | 27.02908 | 127.942 |
|  |  | Okinawa | OkiN | 1 | 1 | 15 | 26.70473 | 128.13371 |
|  |  |  | OkiS | 6 | 6 | - | 26.1705 | 127.8298 |
|  |  | Tonaki | Ton | 10 | 8 | 5 | 26.35752 | 127.15397 |
|  | Southern Ryukyu | Miyako | Miy | 11 | 8 | 6 | 24.7336 | 125.45389 |
|  |  | Tarama | Tar | 11 | 8 | - | 24.67213 | 124.69679 |
|  |  | Ishigaki | Ish 5 | 7 | 3 | 6 | 24.55867 | 124.29066 |
|  |  |  | Ish 6 | 7 | 1 | 6 | 24.60952 | 124.31468 |
|  |  |  | Ish 7 | 7 | 4 | - | 24.46026 | 124.1423 |
|  |  | Iriomote | Iri | 11 | 8 | 11 | 24.43621 | 123.77564 |
|  |  | Yonaguni | Yon 1 | - | - | 8 | 24.44258 | 122.97829 |
|  |  |  | Yon 2 | 8 | 4 | 6 | 24.43893 | 122.96731 |
|  |  |  | Yon 4 | 7 | 4 | - | 24.46186 | 123.03832 |
| *C. taitungensis* | Taiwan | Coastal Mountain Range | CMR | 17 | 7 | 10 | 23.05401 | 121.28706 |
|  |  | Hongye | Hon | 13 | 7 | 10 | 22.86772 | 121.02943 |
| Total | | | | 239 | 110 | 188 |  |  |

**Table S2** Quality information of sequenced library

| Library ID | Total read bases (bp) | Total reads | GC(%) | AT(%) | Q20(%) | Q30(%) |
| --- | --- | --- | --- | --- | --- | --- |
| Library 1 | 19,924,855,888 | 131,952,688 | 48.15 | 51.85 | 96.71 | 92.24 |
| Library 2 | 22,961,655,544 | 152,063,944 | 48.24 | 51.76 | 96.86 | 92.55 |
| Library 3 | 21,613,564,690 | 143,136,190 | 46.42 | 53.58 | 96.67 | 92.19 |
| Library 4 | 23,829,781,724 | 157,813,124 | 48.01 | 51.99 | 97 | 92.86 |
| Library 5 | 22,747,586,770 | 150,646,270 | 47.38 | 52.62 | 96.86 | 92.57 |
| Library 6 | 25,141,040,356 | 166,496,956 | 47.2 | 52.8 | 97.08 | 92.98 |
| Library 7 | 23,543,470,624 | 155,917,024 | 47.02 | 52.98 | 97.27 | 93.5 |
| Library 8 | 22,433,256,412 | 148,564,612 | 47.96 | 52.04 | 97.15 | 93.31 |
| Library 9 | 21,086,640,828 | 139,646,628 | 49.18 | 50.82 | 97.29 | 93.52 |
| Library 10 | 26,901,874,006 | 178,158,106 | 49.69 | 50.31 | 97.27 | 93.46 |
| Library 11 | 24,982,948,188 | 165,449,988 | 50.03 | 49.97 | 97.21 | 93.31 |
| Library 12 | 23,492,416,618 | 155,578,918 | 46.71 | 53.29 | 97.24 | 93.45 |

**Table S3** Prior settings of model parameters in ABC

| **Type** | **Parameter** | **Dist (min,max)** |
| --- | --- | --- |
| integer | N_CR2_ | Unif (50,5000) |
| interger | N_CT2_ | Unif (50,5000) |
| float | N_anc_REL_ | Unif (0.1,10) |
| float | N_CR_REL_ | Unif (0.1,10) |
| float | N_CT_REL_ | Unif (0.1,10) |
| integer | t1 | Logunif (5,3E+5) |
| integer | t2 | Logunif (5,3E+5) |
| integer | t3 | Unif (10,3E+5) |
| float | m_CT1_ | Logunif (1E-9,0.1) |
| float | m_CR1_ | Logunif (1E-9,0.1) |
| float | m_CT2_ | Logunif (1E-9,0.1) |
| float | m_CR2_ | Logunif (1E-9,0.1) |
| float | µ | Unif (2E-9,2E-7) |
| **Rules** |  |  |
| t1 < t2 |  |  |
| t2 < t3 |  |  |

**Table S4** Pairwise Fst of every islands

|  | Kag | Tan | Ama | Ihe | Oki | Ton | Miy | Tar | Ish | Iri | Yon | Fuj |
| --- | --- | --- | --- | --- | --- | --- | --- | --- | --- | --- | --- | --- |
| Tan | 0.048 |  |  |  |  |  |  |  |  |  |  |  |
| Ama | 0.055 | 0.088 |  |  |  |  |  |  |  |  |  |  |
| Ihe | 0.482 | 0.504 | 0.595 |  |  |  |  |  |  |  |  |  |
| Oki | 0.358 | 0.359 | 0.470 | 0.084 |  |  |  |  |  |  |  |  |
| Ton | 0.357 | 0.328 | 0.436 | 0.420 | 0.124 |  |  |  |  |  |  |  |
| Miy | 0.428 | 0.444 | 0.522 | 0.201 | 0.095 | 0.276 |  |  |  |  |  |  |
| Tar | 0.320 | 0.336 | 0.401 | 0.179 | 0.070 | 0.239 | 0.049 |  |  |  |  |  |
| Ish | 0.468 | 0.485 | 0.556 | 0.108 | 0.140 | 0.354 | 0.104 | 0.108 |  |  |  |  |
| Iri | 0.428 | 0.447 | 0.523 | 0.105 | 0.099 | 0.305 | 0.076 | 0.086 | 0.030 |  |  |  |
| Yon | 0.483 | 0.515 | 0.605 | 0.060 | 0.183 | 0.405 | 0.209 | 0.191 | 0.099 | 0.112 |  |  |
| Fuj | 0.050 | 0.067 | 0.177 | 0.298 | 0.110 | 0.129 | 0.222 | 0.117 | 0.279 | 0.217 | 0.316 |  |
| Tai | 0.196 | 0.181 | 0.273 | 0.191 | 0.056 | 0.072 | 0.149 | 0.117 | 0.201 | 0.165 | 0.206 | 0.057 |

**Table S5** The examined specimens

The holotypes are underlined with boldface, islands are underlined, and the collectors are marked in italics.

**CHINA**

Fujian, Fuzhou county, Qi-Yun Ma No 01421, Sep. 1974 (PE 00047527)

**TAIWAN**

**Taitung County:**

Kaede, Kanzan-gun, *Tagawa 3240*, 9 Mar. 1940 (TNS); Peinan, *S. Sasaki s.n.*, 28 May 1920 (TNS 387379); *S. Saito s.n.*, 30 Sep. 1929 (TNS 645694); *S. Sasaki s.n.*, 22 May 1920 (TNS 387374 - TNS 387377, TNS 387380 - TNS 387381); *Tagawa s.n.*, 9 Mar. 1940 (TNS 156738); inter Seisui et Matsuyama, *Y. Yamamoto et. Z. Goto s.n.*, 28 Dec. 1928 (TAI 012507, TAI 194153, TAI 194156, TAI 194157, TAI 194159); Peinan, *S. Sasaki s.n.*, 22 May 1920 (TAI 012505, TAI 012508, TAIF 2561 - TAIF 2564, TAIF 114906 - TAIF 114909); Yengping Hsiang, Taiwan Cycas Nature Reserve, on the trail from the worker`s hut to the Cycas reserve, Broadleaf virgin forest, Exposed cliff or semi-shaded slope at 500 – 800 m a.s.l., *Shu-Chuan Hsiao 1251*, 17 May 1995 (HAST 53089); Yengping Hsiang, Cycas Reserve, On a steep mountain slope (steepness ca. 45) well exposed, in a secondary forest mixed with *Cyclobalanopsis glauca*, *Lagerstroemia subcostata*, *Zelkova serrata* etc., at 500 m a.s.l., *Chih-Hua Tsou 825*, 28 Jun. 1993 (**HAST 63691**); near Kaede, Kwanzan-gun, *M. Tagawa s.n.*, 9 Mar. 1940, (KYO 3240); Luye, *Kuo-Shih Hsu s.n.*, 20 Sep. 1994 (PE 00019780, PE 00019781); Honye, *Chih-Hua Tsou s.n.,* Sep. 1992 (PE 01901142); Peinan, *S. Sasaki*, 25 Feb. 1925 (TAIF 114913, TAIF 114915 - TAIF 114917)

**JAPAN**

**Unknown Area:** *C. P. Thunberg*, 1775 (UPS 23733, **UPS 23734**, UPS 23735)

**Miyazaki Prefecture:**

Toi cape, *Okuyama 17075*, 27 Mar. 1958 (TNS 274967); Toi cape, *Satoo Kuri s.n.*, Dec 1942 (TNS 796860); Toi, *Hayakawa s.n.*, 2 Apr. 1924 (TI 00049161, TI 00049162); Cape Toi, *S. Saito s.n.*, 2 Apr. 1924 (TI 00049168)

**Kagoshima Prefecture:**

Satsuma Peninsula, Yamakawa-Nagasakihana, *S. Saito s.n.*, 18 Oct. 1928 (TI 00049164); Hizaki, *Furusawa Isao s.n.*, 4 Aug. 1946 (TI 00049177, TI 00049169, TI 00049178, TI 00049185); Akamisubana, Yamakawa-Maehi, 20 – 50 m a.s.l., *M. Togashi, G. Murata & H. Kanai s.n.*, 23 Jan 1965 (TI 00049188, TI 00049189); Nagasakihana, Yamakawa-cho, Ibusuki-gun, *G. Murata s.n., M. Togashi & H. Kanai*, 23 Jan 1965 (KYO 111); Koneshime Kimotsuki-gun Prov. Ohsumi, *Tomitaro Makino s.n.*, Aug. 1910 (KYO 12510); Ohsumi, between Oodomari and Satamisaki, Satatyo, *G. Murata s.n.*, 10 Aug. 1959 (KYO 13025); Ohsumi, Satamisaki, *S. Kitamura s.n.*, 20 Jul. 1944 (KYO s.n.); Satsuma prov., *G. Koizumi s.n.*,10 Mar. 1940 (KYO); Hizaki, *Furusawa, Isao* *s.n.*,4 Aug. 1946 (TI 00049175)

Tanegashima: Nishino-omote, *J. Ohwi & K. Okamoto*, 7 Nov. 1953, TSM. No. 994 (PE 00047259, PE 00039712, PE 00039726, PE 00036574, PE 00047260, PE 00038183, TI 00049176, KYO 50427, TNS 106684); Minamitane-cho, Kukinaga, sandy beach, grassland, sunny, dried, abundant at 13 m a.s.l., *Goro Kokubugata 15539*, 24 Feb. 2013 (TNS 01183883)

Takarajima: 30 – 50 m a.s.l., *T. Shiuchi s.n.*, 16 May 1993, TAK 1454 (KAG)

Kodakarajima: 10 m a.s.l., *T. Shiuchi s.n.*, 21 May 1993, TAK 1693 (KAG)

Akusekijima: 110 - 200 m a.s.l., *T. Shiuchi s.n.*, 8 Jul. 1994, AKU 5185 (KAG)

Kikajima: *S. Tsugaru 25387*, 8 Aug. 1997 (TNS 659085)

Amami: at 105 m a.s.l., *S. Tsugaru* *33830*, 27 Jul. 2005 (KYO); at 100 m a.s.l., *S. Tsugaru, Harumichi Murakami & Idzumi Matsuka* *No 20683*, 3 Aug. 1994 (KYO); interior of Kawauchi, Sumiyo-mura, on exposed fields, *K. Iwatsuki*, 20 Aug. 1959, No 5035 (KYO); Yoro Shima Isl. Central-south part, *Suzuki Eizi s.n.*, 21 May 2018 (KAG 019468); *Soma s.n.*, 11 Apr. 1970 (TNS 01047388); *S. Tsugaru 33830*, 27 Jul 2005 (TNS); *S. Tsugaru 20683*, 3 Aug 1994 (TNS); Naze, *Furusawa Isao s.n.*, 13 Sep. 1940 (TI 00049183)

Tokunoshima: between Omo and Nanbaru, Tokunoshima-cho at 100-128 m a.s.l., along road on the small hill on coral reef, *G. Murata s.n.*, 11 Mar. 1986, No 56241 (KYO 00050418 - KYO 00050420)

**Okinawa Prefecture:**

Okinawa: *Ito s.n.*, 28 Jul. 1894 (TNS 60832); *Tokutaro Ito s.n.*, 28 Jul 1894 (TNS 382664, TNS 382665); rocky seaside, sunny and dried, abundant, *Goro Kokubugata 7147*, 5 Dec. 2005 (TNS); natural trail near Gushikamia Gymnasium, under a broad leaves forest, slight sunny, slight dried, abundant at 49 m a.s.l., *Goro Kokubugata* *7146*, 5 Dec. 2005 (TNS); Chinen Cape, Kudeken, Sashiki-cho, under a broad leaves forest, slight sunny, slight dried, abundant at 59 m a.s.l., *Goro Kokubugata 7145*, 5 Dec. 2005 (TNS); near Mt. Kongosekisai-yama, Hedo, Kunigami-son*,* under a broad leaves forest, slight sunny, moist at abundant 121 m a.s.l., *Goro Kokubugata 7142*, 4 Dec. 2005 (TNS); Uehara, Miyagi-jima, Yonashiro-cho*,* under a broad leaves forest, slight sunny, dried, abundant at 86 m a.s.l., *Goro Kokubugata 7144*, 4 Dec. 2005 (TNS); near lighthouse in northern cape, Ikei-jima, Yonashiro-cho, rocky seaside, sunny, abundant, at 20 m a.s.l., *Goro Kokubugata 7143*, 4 Dec. 2005 (TNS); side of R58, 1km N from Nago-shi, Oogimi-son, on rock in the sea side, sunny, dried, abundant, *Goro Kokubugata 1373*, 22 May 2002 (TNS); side of R58, 1 km N from Nago-shi, Oogimi-son, on rock in the sea side, sunny, dried, abundant, *Goro Kokubugata 1372*, 22 May 2002 (TNS); 300 m W from Root 58, Uka, Kunigami-son, under a broad leaves forest, slight sunny, moist, abundant, *Goro Kokubugata 7136*, 4 Dec. 2005 (TNS); coastal Hwy 10 m away from the sea, broadleaf forest, winter pine & *Cycas*, at 5 m a.s.l., Ching-I Peng 15547, 25 Jun. 1993 (HAST 81445, HAST 81446); south foot of Mt. Hedozaki-yama, Kunigami-son, Kunigami-gun, on calcareous rocky slope at 100 m a.s.l., *G. Murata s.n., Hiroshige Koyama & Aya Nitta s.n.*, 16 Oct. 1986, No 56834 (KYO 00050417); Mt. Hedozaki, Hedo Kunigami-gun, *K. Iwatsuki, C. Phengklai, M. Wakabayashi & M. Kato s.n.*, 28 Oct. 1973, No 309 (KYO 00050421, KYO 00050422); Oku, *M. Tamura 24854*, 9 Oct. 1972 (PE 00001737)

Iheya: rocky seaside, heavy sunny, heavy dried, abundant, at 5 m a.s.l., *Goro Kokubugata 14531*, 10 Feb. 2012 (TNS)

Miyako: Hiragama, *Suguki s.n.*, 14 Dec. 1937 (TAI 012501); *G. Masamune s.n.*, 1 Aug. 1934 (TAI 119967)

Iriomote: Komi to Funaura, Taketomi-cho, Yayeyama-gun, along road near sea coast, *G. Murata, Hiroshige Koyama & Aya Nitta s.n.*, 10 Oct. 1986, No 67566 (KYO 00050424); Komi to Funaura, Taketomi-cho, Yayeyama-gun, along road near sea coast, *G. Murata, Hiroshige Koyama & Aya Nitta s.n.*, 10 Oct. 1986, No 67525 (KYO 00050426)

Ishigaki: *G. Masamune & S. Suzuki s.n.*, 3 Jul 1935 (TAI 119968)

Yonaguni: Yonaguni, Higawa, *S. Hatusima, K. Shimabuku, Y. Miyagi, E. Nakata & M. Furuse*, 1 Oct. 1973 (TNS 01167335 - TNS 01167339, TNS 01167341


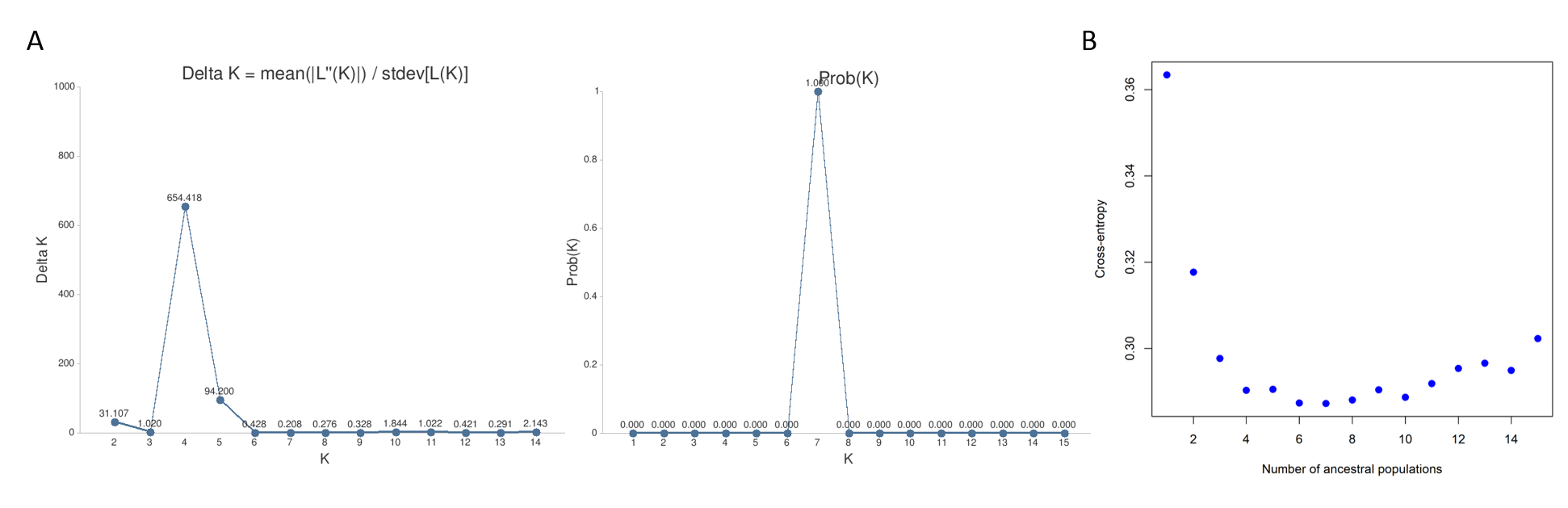


Fig. S1 Results of (A) Evanno`s method in StrAuto and (B) cross-entropy in sNMF


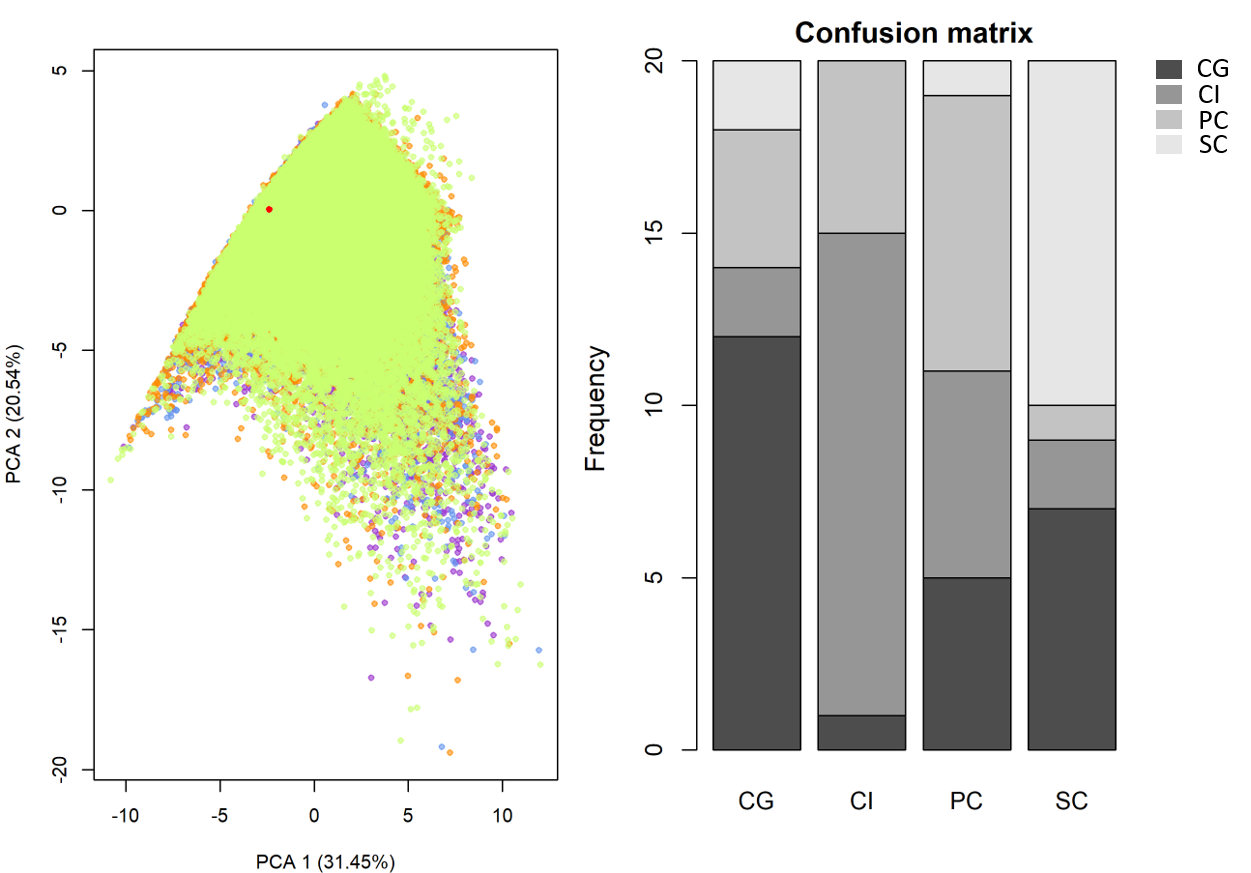


Fig. S2 PCA of the summary statistics in the four speciation models in ABC and their model validation after collinearity removal.
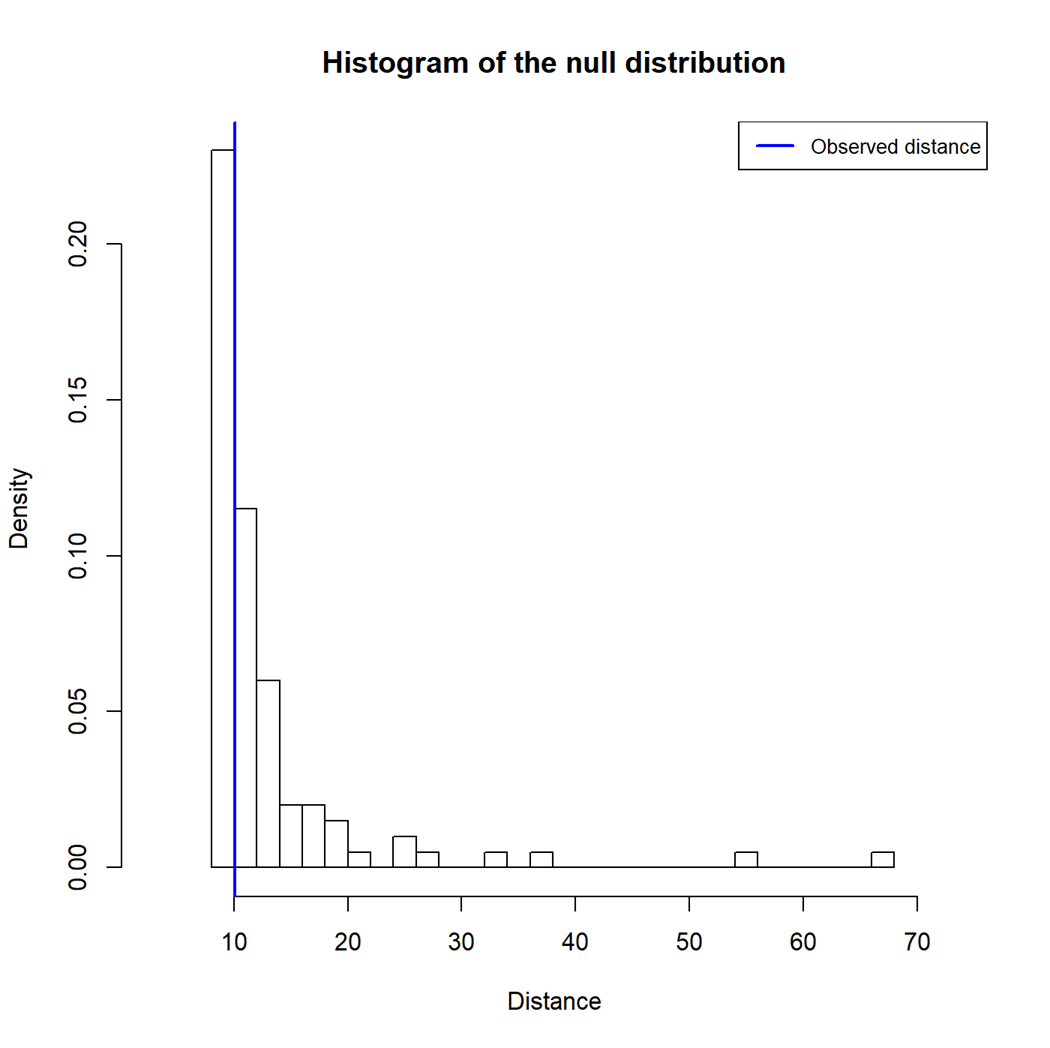


Fig. S3 Goodness-of-test of simulation data against the observed values.
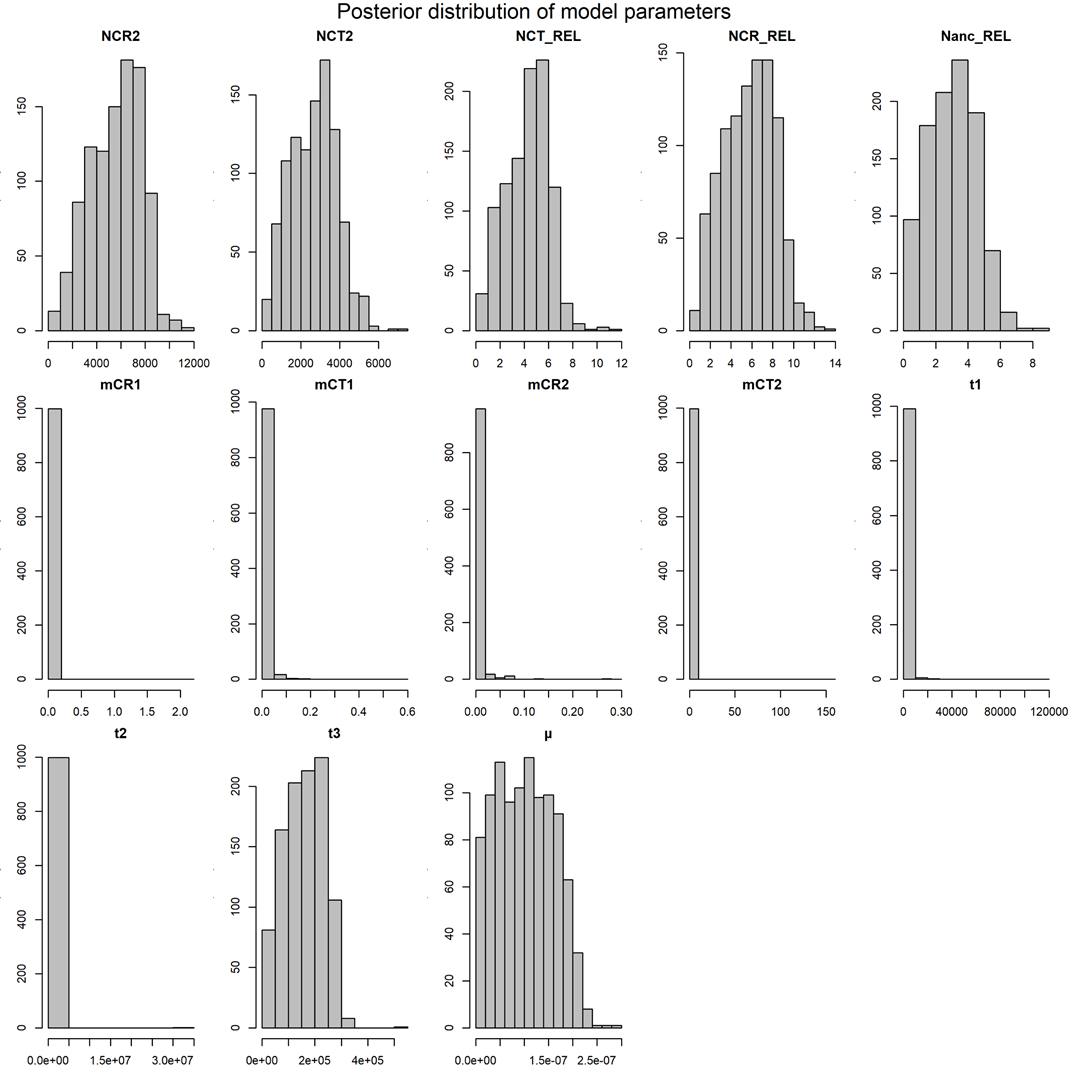


Fig. S4 Posterior distribution of the model parameters in the best model.


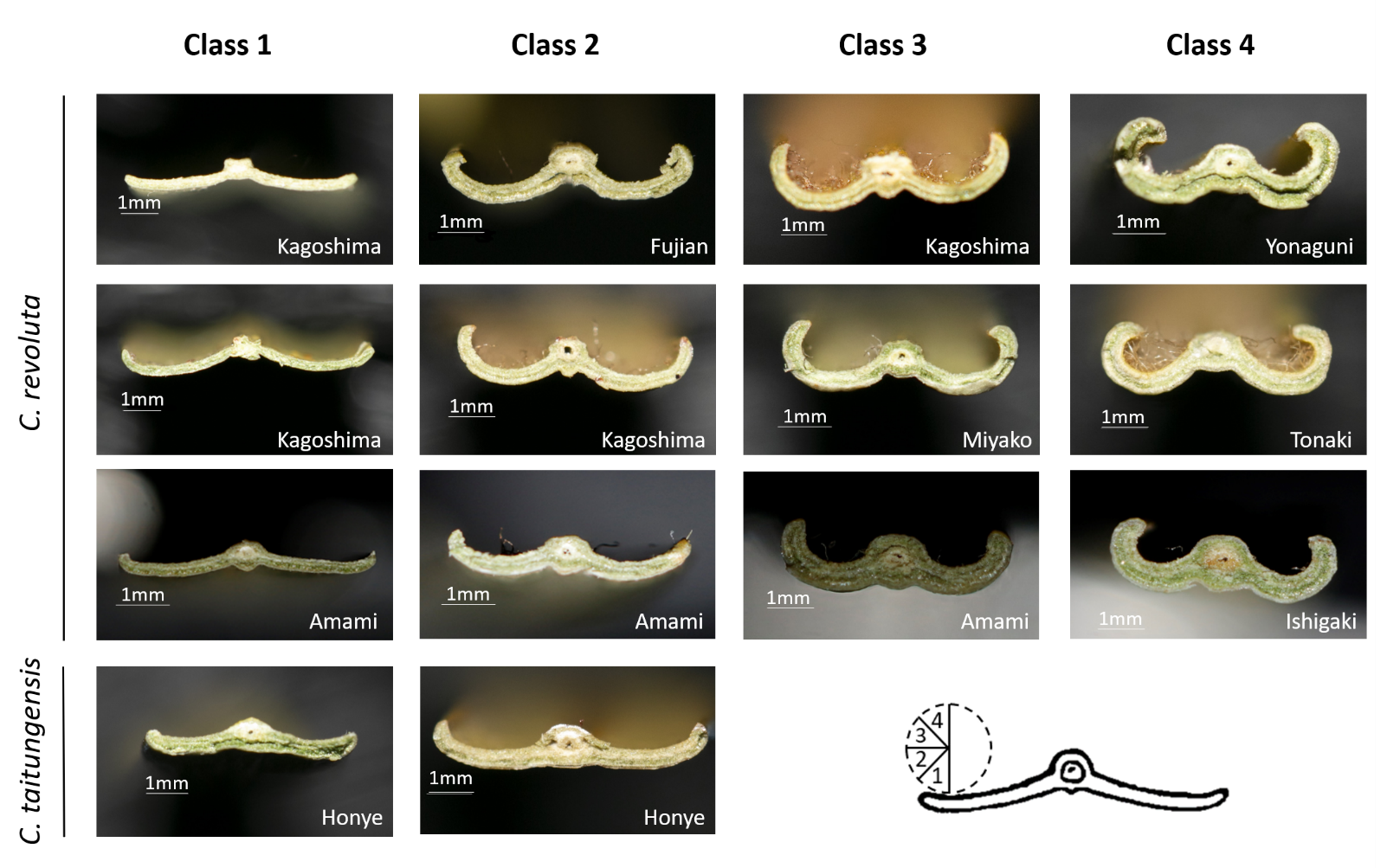


Fig. S5 Cross section of leaflet lamina in two species.


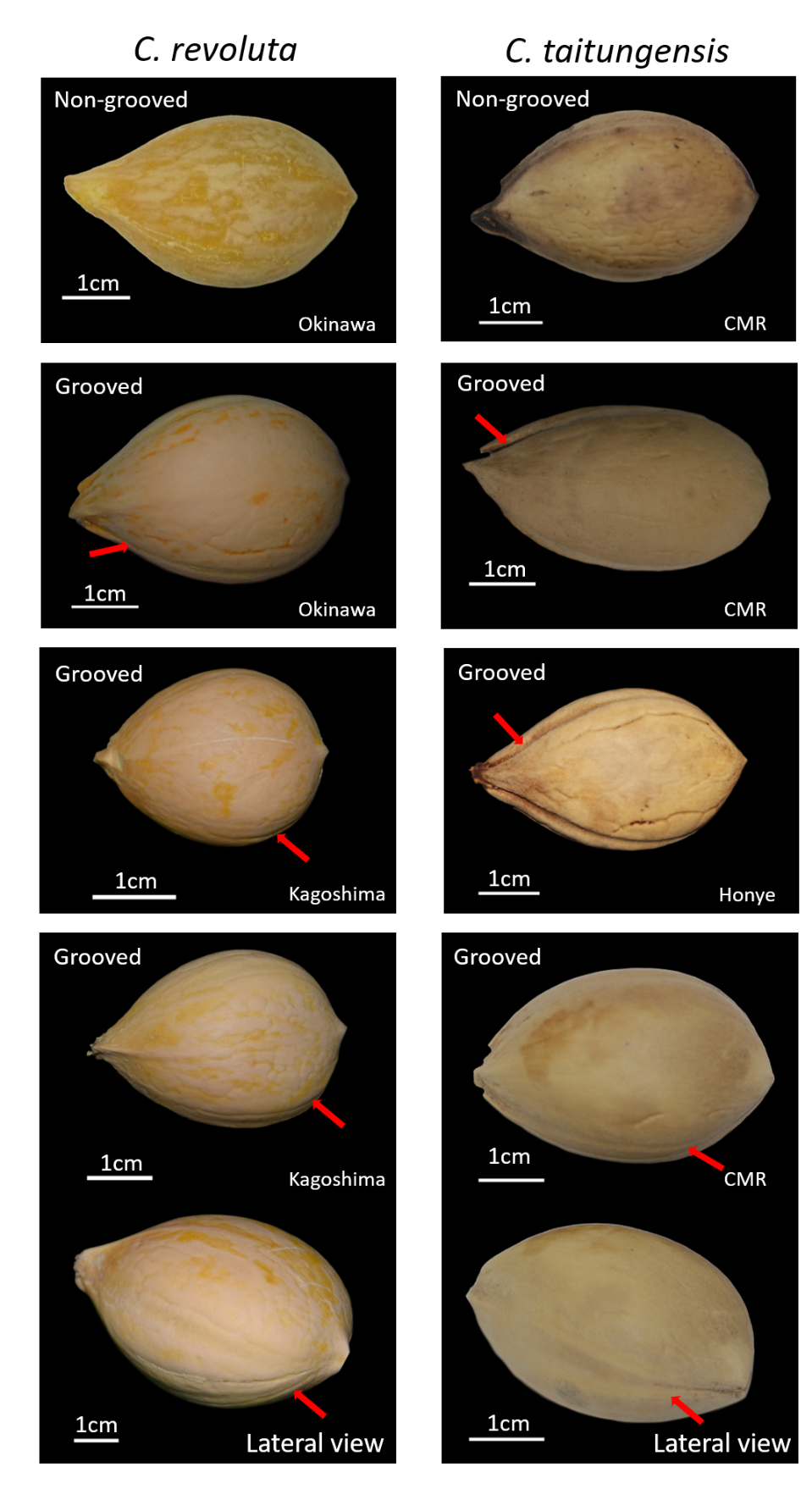


Fig. S6 Sclerotesta groove in two species. The red arrow point to the position of groove.


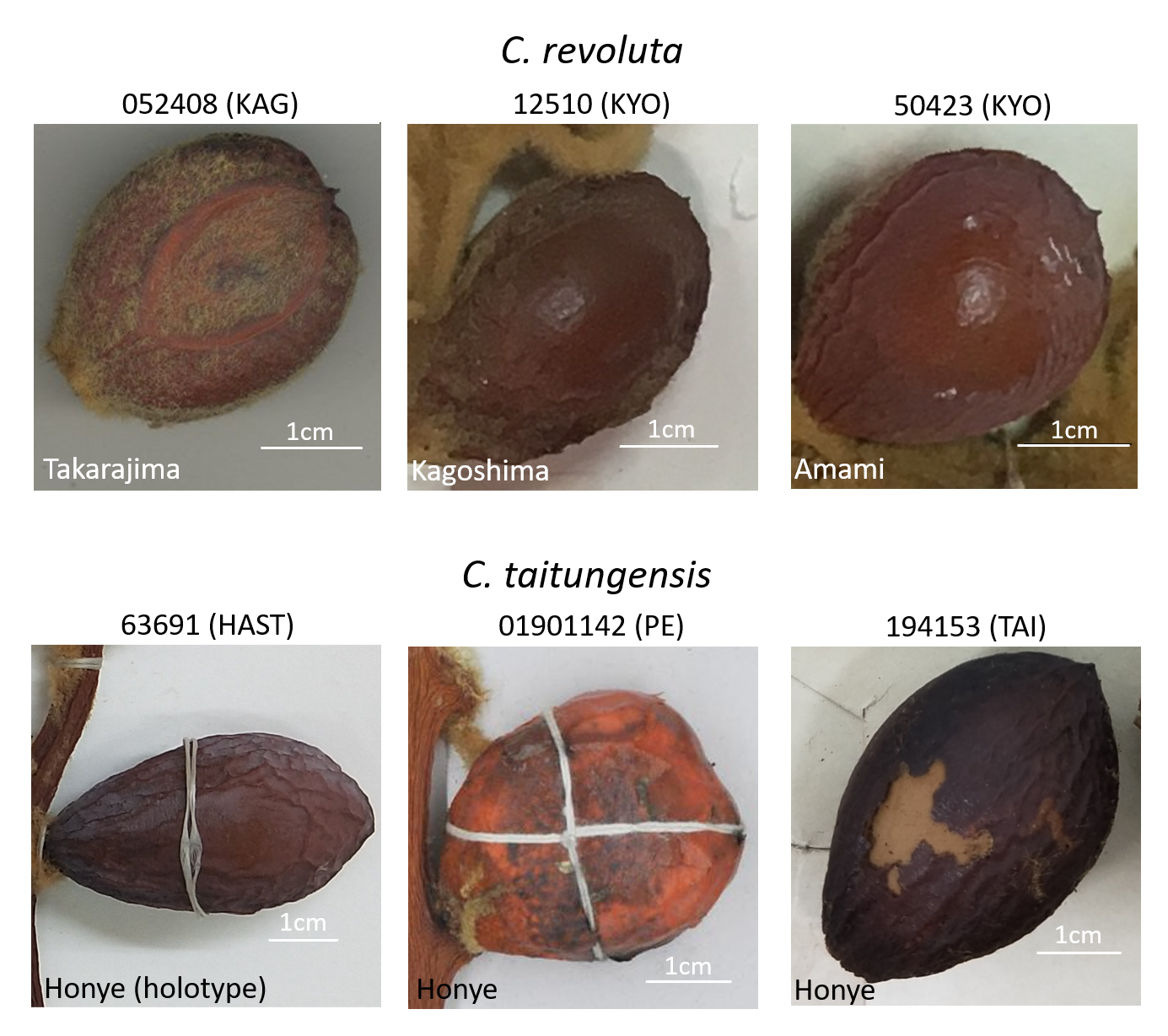


Fig. S7 Seed morphology and sarcotesta color in two species.


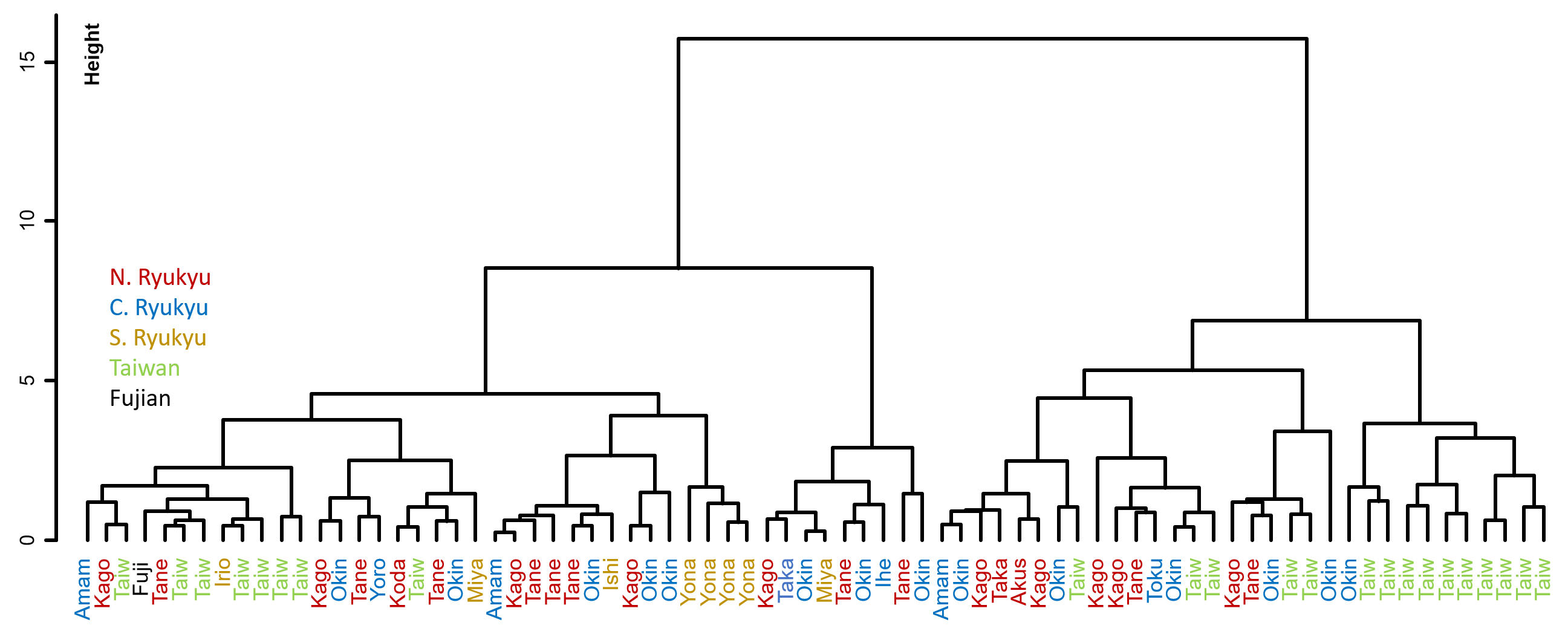


Fig. S8 Dendrogram of significant leaflet traits in Fig. 3.

**References**

Charlesworth, B. (2009). Fundamental concepts in genetics: effective population size and patterns of molecular evolution and variation. *Nat Rev Genet* 10**,** 195-205.

De La Torre, A.R., Li, Z., Van De Peer, Y., and Ingvarsson, P.K. (2017). Contrasting Rates of Molecular Evolution and Patterns of Selection among Gymnosperms and Flowering Plants. *Mol Biol Evol* 34**,** 1363-1377.

Frankham, R. (1995). Effective population size/adult population size ratios in wildlife: a review. *Genetics Research* 66**,** 95-107.

Frankham, R., Bradshaw, C.J.A., and Brook, B.W. (2014). Genetics in conservation management: Revised recommendations for the 50/500 rules, Red List criteria and population viability analyses. *Biological Conservation* 170**,** 56-63.

Hanlon, V.C.T., Otto, S.P., and Aitken, S.N. (2019). Somatic mutations substantially increase the per-generation mutation rate in the conifer Picea sitchensis. *Evolution Letter* 3**,** 348-358.

Nagalingum, N., Marshall, C., Quental, T., Rai, H., Little, D., and Mathews, S. (2011). Recent synchronous radiation of a living fossil. *Science* 334**,** 796-799.

Wegmann, D., Leuenberger, C., and Excoffier, L. (2009). Using abctoolbox. *BMC Bioinformatics* 11**,** 116.

Zheng, Y., Liu, J., Feng, X., and Gong, X. (2017). The distribution, diversity, and conservation status of Cycas in China. *Ecol. Evol.* 7**,** 3212-3224.
